# Supplementary material for: Sustainment of a patient flow intervention in an intensive care unit in a regional hospital in Australia: a mixed-method, 5-year follow-up study
Source: BMJ Open. 2021 Jun 21;11(6):e047394. doi: 10.1136/bmjopen-2020-047394 (PMC8220473; doi:10.1136/bmjopen-2020-047394)
Supplement: Supplementary data [file bmjopen-2020-047394supp001.pdf]

Appendix A: Interview Schedule

The information collected will be demographic information and responses to the following questions.

A: The ICU Escalation plan and morning handover meeting implementation

Do you know of the ICU Escalation plan and morning handover meetings that was implemented 2014?
Were you involved in the implementation process?
If so – what was your role?
What is your experience of the morning handover meetings (if relevant)?
What is your experience of the ICU Escalation plan (if relevant)?
Can you describe an episode when the ICU Escalation plan was activated (if relevant)?

B: ICU Resource allocation processes

Please describe how beds and resources are allocated in the ICU.
What positions have an input into these decisions?
What are the factors involved?
Does the process change as the ICU becomes busier?

C: Work practice changes

Questions to the hospital management group.
How does your work at the hospital involve the ICU?
Tell me how the ICU impacts on your daily work routine.
Are you aware of any change to your work practice due to the Escalation plan implementation?
Are you aware of any change to the work practices of other staff as a result of the initiatives?

Questions to ICU staff.
Are you aware of any change to your work practice due to the morning handover meetings?
Are you aware of any change to your work practice due to the Escalation plan implementation?
Are you aware of any change to the work practices of other staff as a result of the initiatives?

D: Job satisfaction

How do you feel about coming to work each day, particularly on days with heavy ICU involvement?
Was there any impact of the initiatives on your job satisfaction?
Did the initiatives make any difference to the days when ICU resources where stretched?
Do you feel patient care was improved?

E: Questions on the sustainability of the ICU Escalation Plan and daily morning meeting. Two different sets of questions to be used depending on the results of the analysis of the quantitative data.

I) *If the analysis of data shows that the improvement has sustained over time*
To your opinion – why has the benefits of the invention sustained over time?
What has been the key factors?
Who has been the key players? What did they do to help the success?
What suggestions do you have for the work with optimizing flow of patients through ICU?

II) *If the analysis of data shows that the improvement has not sustained over time*
To your opinion – why has the benefits of the invention not sustained over time?
What has been the obstacles?
What could have been done differently?
What suggestions do you have for the work with optimizing flow of patients through ICU?

F: Demographic Information

This information will not be linked to participants name. A number will be given to be used as a code reference for analysis purposes.

1.Gender
2.Profession
3.Role at TTHS
4.Time since qualification to practice his profession
5.Time in this organisation
6.Time in ICU
