# Supplementary material for: Sustainment of a patient flow intervention in an intensive care unit in a regional hospital in Australia: a mixed-method, 5-year follow-up study
Source: BMJ Open. 2021 Jun 21;11(6):e047394. doi: 10.1136/bmjopen-2020-047394 (PMC8220473; doi:10.1136/bmjopen-2020-047394)
Supplement: Supplementary data [file bmjopen-2020-047394supp002.pdf]

Appendix B: Code schedule

**Theme**	**Sub-theme**	**Code**	**Quotes**
Sustained benefits of implementation	Positive experiences	Facilitates communication within microsystem	Nurse 3: *"[…] as soon as you might say to one of the intensivists, where are we, and you can say, red, they know exactly the situation we're in. So, it's a good, quick identification."*
		Work-as-imagined reconciles work-as-done	Nurse 2: *"The doctors have been really good, so then they won't admit or […] they wouldn't admit high dependency patients. […] They've been very good supporting the staff and they've been - at one stage I remember them actually having to transfer to The Mater or something patients that had come into ED (emergency department), because we couldn't take them here, because he had no beds."*
		Improves teamwork	Manager 1: *"I think the morning meetings have made everybody more aware and tried to collaborate as a team approach, and I think that's really important."*
	Enabling factors	Morning meeting setting benefits	Manager 1: *"[…] it is continuous but that is the only point in time you can have everybody to sit down."*
		Everyone was invested	Doctor 4: *"Well, it worked because it was something everyone was willing to try."*
Factors adversely affecting sustainability	Management of operations	Leadership not understanding micro level work	Doctor 3*: "I'm going to be harsh here, a lot of the senior nursing staff who are going to be making those decisions are not there at ten o'clock at night or two in the morning, having to actually wear the brunt, the consequences of those decisions."*

		Hierarchical barriers to improvement	Nurse 2: *"[…] sometimes there are good leaders and there are bad leaders and there are good leaders that help people progress and things like that. Then there are some people that just put barriers in front of people. There are some people that just like arguing for the sake of arguing, which doesn't do the unit very good."*
		Pressure for elective surgeries	Doctor 1: *"There's a massive push to meet our NEST targets and we are behind and our service group is, I think, the only one who ended up in the red financially because we didn't meet our targets."*
		Lack of ICU resources	Manager 3*: "[…] if we have the same amount of ICU beds next year, the number of elective surgeries we cancel will just increase. If there's no more money for ICU then we just have to accept that there's going to be more cancellations and longer delays, that's reality."*
	Lack of communication and understanding	Lack of knowledge about implementation	Nurse 3: *"I don't know whether the new registrars and the new shift coordinators have a full grasp of the traffic light system."*
		Micro doesn't understand the escalation plan	Doctor 2: *"At the borderline state it doesn't do a lot. When it's really severe, when reach beyond red, which is very rare, it could be useful."*
		Lack of communication and discrepancy within microsystem	Doctor 1: *"[…] there has been loss of transparency about the staffing that we do have in the unit. We are often told oh, don't worry about that or we'll get staff, or you don't need to know that or yep, so I think that the lack of transparency and that actual*

			discussion about where we are, has been lost."
		Lack of communication and discrepancy between microsystems	Nurse 1: *"They have different booking staff down there that don't really know what goes on up here and so they'll just have a surgeon that wants to operate. He's got a theatre, so they'll say yes to anything not really realising that those patients do need to come to an intensive care unit […]."*
		Lack of communication between micro and meso	Doctor 2: *"[…] ICU need to speak the same language with the executive and surgical service group managers. We are not speaking the same language."*
	Plans not working as intended	Misuse of traffic light	Manager 2: *"There's a lot of disagreement, I think, around even though we've all agreed on the criteria for red, there's still, on the day of, flexibility in the thinking that yes you can get that heart done or yes you can get that elective surgery case in or no you can't, that sort of give and take."*
		Use of escalation plan has changed over time	Doctor 3: *"The process at the beginning was fine. It's been progressive reinterpretation that's given it grief."*
		Extra effort needed	Nurse 4: *"There are many days where we haven't get breaks. We just don't get breaks. We can't get staff to the bedside to help with turns […]. You should have two nurses to turn them. You don't do that. You can't get them. People get cranky with each other because they're not getting their breaks. We get around things by - often - I can't count the number of times I have brought my food to the bedside to eat[…]."*

		Consequences of over-loaded ICU	Doctor 4*: "[…] we couldn't bring the patient to ICU because there was no nurse to look after the patient. That actually made me go sick. […]I actually took time off sick, stress leave, and developed shingles, reactivation of chickenpox due to the stress."*
		Implementation not comprehensive enough	Manager 2: *"[…] it's very difficult to draft for everything that could happen and it's not black and white"*
	Reality of clinical work	Disturbance in patient flow	Manager 4: *"And then there's days where there's, potentially, patients who could go to the ward, and there's nowhere to send them to."*
		Increased workload for nurses	Nurse 2: *"I think the workload's increased for the nurses."*
		Staffing problem	Nurse 2: *"We have more new nurses. […] For transition. Like, they've just gone to uni. They've had no ward - they've had no experience whatsoever and they're coming up here, so that takes a lot of time away."*
		New clinical and administrative processes	Doctor 2*: "So it's actually worse in the last year with the EMR (electronic medical record) because it increased our workload."*
		Natural variation	Doctor 1: *"[…] January and February are classically - and I think it happens every year, really slow months. Then around August is always a peak. It's around flu season and more people have MIs and things like that but it seems to be a - not*

			necessarily predictable but a constant variation."
