# Supplementary material for: Sustainment of a patient flow intervention in an intensive care unit in a regional hospital in Australia: a mixed-method, 5-year follow-up study
Source: BMJ Open. 2021 Jun 21;11(6):e047394. doi: 10.1136/bmjopen-2020-047394 (PMC8220473; doi:10.1136/bmjopen-2020-047394)
Supplement: Author's manuscript [file bmjopen-2020-047394.draft_revisions.pdf]

BMJ Open is committed to open peer review. As part of this commitment we make the peer review history of every article we publish publicly available.

When an article is published we post the peer reviewers' comments and the authors' responses online. We also post the versions of the paper that were used during peer review. These are the versions that the peer review comments apply to.

The versions of the paper that follow are the versions that were submitted during the peer review process. They are not the versions of record or the final published versions. They should not be cited or distributed as the published version of this manuscript.

BMJ Open is an open access journal and the full, final, typeset and author-corrected version of record of the manuscript is available on our site with no access controls, subscription charges or pay-per-view fees (http://bmjopen.bmj.com).

If you have any questions on BMJ Open's open peer review process please email info.bmjopen@bmj.com

BMJ Open

A multi-method study on the sustainment of an implementation: An ICU patient flow intervention revisited

Journal:	*BMJ Open*
Manuscript ID	bmjopen-2020-047394
Article Type:	Original research
Date Submitted by the Author:	30-Nov-2020
Complete List of Authors:	Ros, Eva; Linköping University, Department of Biomedical and Clinical Sciences; Macquarie University Australian Institute of Health Innovation<br>Ros, Axel; Jönköping University, Jönköping Academy for Improvement of Health and Welfare, The School of Health and Welfare, ; Macquarie University Australian Institute of Health Innovation<br>Austin, Elizabeth; Macquarie University, Australian Institute of Health Innovation<br>De Geer, Lina; Linköping University Hospital, Department of Anaesthesiology and Intensive Care ; Linköping University, Department of Biomedical and Clinical Sciences<br>Lane, Paul; Townsville Hospital and Health Service<br>Johnson, Andrew; Townsville Hospital and Health Service; James Cook University College of Medicine and Dentistry<br>Clay-Williams, Robyn; Macquarie University, Australian Institute of Health Innovation
Keywords:	Health & safety < HEALTH SERVICES ADMINISTRATION & MANAGEMENT, Quality in health care < HEALTH SERVICES ADMINISTRATION & MANAGEMENT, Adult intensive & critical care < INTENSIVE & CRITICAL CARE, QUALITATIVE RESEARCH

SCHOLARONE™
Manuscripts

I, the Submitting Author has the right to grant and does grant on behalf of all authors of the Work (as defined in the below author licence), an exclusive licence and/or a non-exclusive licence for contributions from authors who are: i) UK Crown employees; ii) where BMJ has agreed a CC-BY licence shall apply, and/or iii) in accordance with the terms applicable for US Federal Government officers or employees acting as part of their official duties; on a worldwide, perpetual, irrevocable, royalty-free basis to BMJ Publishing Group Ltd ("BMJ") its licensees and where the relevant Journal is co-owned by BMJ to the co-owners of the Journal, to publish the Work in this journal and any other BMJ products and to exploit all rights, as set out in our licence.

The Submitting Author accepts and understands that any supply made under these terms is made by BMJ to the Submitting Author unless you are acting as an employee on behalf of your employer or a postgraduate student of an affiliated institution which is paying any applicable article publishing charge ("APC") for Open Access articles. Where the Submitting Author wishes to make the Work available on an Open Access basis (and intends to pay the relevant APC), the terms of reuse of such Open Access shall be governed by a Creative Commons licence – details of these licences and which Creative Commons licence will apply to this Work are set out in our licence referred to above.

Other than as permitted in any relevant BMJ Author's Self Archiving Policies, I confirm this Work has not been accepted for publication elsewhere, is not being considered for publication elsewhere and does not duplicate material already published. I confirm all authors consent to publication of this Work and authorise the granting of this licence.

For peer review only

A multi-method study on the sustainment of an implementation: An ICU patient flow intervention revisited

Author Eva Ros 1) 2)
Co-authors Axel Ros 3) 2), Elizabeth E. Austin 2), Lina De Geer 4) 1), Paul Lane 5), Andrew Johnson 5) 6), Robyn Clay-Williams 2).

1) Department of Biomedical and Clinical Sciences, Linköping University, Linköping, Sweden

2) Australian Institute of Health Innovation, Macquarie University, Sydney, Australia

3) Jönköping Academy for Improvement of Health and Welfare, The School of Health and Welfare, Jonkoping University, Jonkoping, Sweden

4) Department of Anaesthesiology and Intensive Care in Linkoping, Sweden

5) Townsville Hospital and Health Service, Townsville, Australia

6) College of Medicine and Dentistry, James Cook University, Townsville, Australia

Corresponding author:
Eva Ros
Vattengatan 36
60220 Norrkoping
Sweden

E-mail: evaro626@liu.student.se
Phone: +4673-525 17 41
Fax number: No fax number available

Keywords: implementation science, change management, multi-method study, quality of health care, intensive care units

Word count: 4074

For peer review only

For peer review only

Abstract

Objective

In 2014 an escalation plan and morning handover meetings were implemented in an Intensive Care Unit (ICU) to reduce access block for post-operative care. This study aimed to revisit the improvement intervention five years on, to see if the changes have been sustained and to understand factors contributing to sustainability.

Design

A multi-method approach was used, involving quantitative analysis of ICU administrative data from 2014 to 2019, and qualitative analysis of interviews with hospital management and ICU staff.

Setting

ICU in a regional hospital in Australia.

Participants

12 interview participants from three groups: ICU nurses, ICU doctors and hospital management.

Main outcome measures

ICU administrative data: monthly number of elective surgeries cancelled due to unavailability of ICU beds, number of planned surgeries, bed status, a workload measure, occupancy and length of stay. Staff perceptions of the interventions and factors contributing to sustainability.

Results

More elective surgeries are cancelled due to unavailability of ICU beds than before the intervention (X^2=16.38, *p*=.003), despite a reduction in the first period after the implementation. Factors contributing to the improvement not being sustained include competitive interests in the management of patient flow, deficiencies in understanding the reasoning behind the interventions and in communication in the ICU and between ICU and other departments. There are discrepancies between how nurses and doctors use the escalation plan and regard the availability of ICU beds.

Conclusion

Improvement interventions in health care that appear initially to be successful are not necessarily sustained over time, as was the case in this study. In a complex adaptive system, there is no such thing as a ‘fix and forget’ solution for interventions as they need to be

For peer review only

flexible to accommodate the dynamics in the conditions for daily work. This has implications for change and change management across healthcare systems.

Strength and limitations of this study

- This is a long-term follow up study of an implementation of an intervention.
- A multi-method approach with both quantitative and qualitative analysis made it possible to both understand if the implementations had sustained or not and describe why it had or had not sustained.
- The multi-method approach used also made it possible to guide the interviews from the quantitative results, but also to verify some of what has been stated in interviews with the quantitative results.
- In the regression analysis the R^2 is small for most analyses which suggests that the independent variable is only explaining a part of the variance in the outcome variable.

Background

The long-term sustainability of interventions implemented in healthcare is important, but seldom evaluated. More typically, the effectiveness of an intervention is assessed shortly after implementation and then the evaluation is deemed to be complete.[1] Single interventions or one-off implementation of an intervention bundle may seem attractive, but they are largely unsuccessful in effecting meaningful change over time in clinical practice.[2] The definition of sustainability is somewhat controversial.[1, 3] It can be described as something steady that does not revert back, but also as a dynamic, fluid moving state where the intervention can adapt to new situations.[4, 5] From a quality and patient safety viewpoint, the importance of an intervention should be the effect of it, not the intervention itself. Therefore, the implementation of an intervention should be considered to be sustainable when the effects of the intervention have been sustained.[1]

Bed occupancy at Intensive Care Units (ICU) can show unexpected large variations due to admission of critical patients. It is therefore a challenge to balance the capacity for planned elective surgeries requiring intensive care after surgery with the unplanned critical patients in need of intensive care, particularly in hospitals with a single ICU for both categories of patients. In 2014, senior clinical staff at the ICU at a regional hospital in Australia saw a need

to optimise the flow of patients through the ICU in order to decrease cancellations of elective surgeries due to the unavailability of ICU beds.[6] An ICU escalation plan, a daily multidisciplinary morning handover meeting, and optional education in system resilience for staff members was developed and implemented after approval of hospital management. The escalation plan, based on three different states – green, amber and red, was introduced as a system to indicate the availability of beds, referred to as the traffic light system (Figure 1). After a follow up period of 10 months the intervention was found to have succeeded, since patient flow improved and elective surgeries cancelled due to the unavailability of ICU beds was significantly reduced.[6] Improved internal communication, decision-making and cohesion within the ICU and better coordination between ICU and other hospital departments, such as surgery and the emergency department was also found.

Healthcare can be described as a complex adaptive system (CAS) with micro-, meso- and macrosystems.[7, 8] In CAS, it is not enough to understand the individual components of a system since that does not help in understanding the system as a whole. What is important is to understand how the individual components are connected and how they adjust to each other, as the interplay between components of a CAS affect system performance. Microsystems are the smaller units that are formed around the patient at the point where patient care is delivered.[9, 10] The different microsystems, for example an ICU or a surgical ward, are connected to each other through the mesosystem. Through this mesosystem the different microsystems can interact and affect each other. The macrosystem regulates the whole healthcare system and its meso- and microsystems through legislation, political prioritisation and national policies.

It is important that improvements in healthcare are sustainable. Yet we know that practices in healthcare often revert back to previous practice once the energy and funding associated with the original intervention are removed.[1] A review on empirical studies of program sustainability found that only 40-60% of interventions are sustained over time.[11] Hence, it is critical to study the sustainability of interventions in addition to the immediate outcomes. The current study revisited the implementation of an ICU escalation plan and morning meeting at the same regional hospital five years on, with the aims:

(1) To find if the effect of the 2014 implementation has been sustained over time or not, and
(2) To understand why the effect of implementation has been sustained or not.

For peer review only

Method

We conducted a multi-method study, collecting both quantitative and qualitative data to examine the sustainability of the intervention over time and staff perspectives regarding the intervention's sustainability.

Quantitative method

Descriptive statistics (number, percentages) were used to tabulate results. SPSS (V27) was used for statistical analysis. ICU audit data was collected from July 2014 to December 2019, where the data from July 2014 to August 2015 was used in the original study.[6] The audit data included monthly number of cancelled surgeries due to no availability of ICU beds, monthly number of planned elective surgeries demanding ICU beds, bed status (green, amber or red based on a traffic light system), nursing Hours Per Patient Day (HPPD; a measure of workload for nurses),[12] bed occupancy and LoS. Bed status was determined from notes recorded at the ICU morning meetings. However, data was incomplete from October 2016 to May 2018 and completely missing from July 2017 to February 2018. The bed status data from October 2016 to May 2018 was collected from referrals, refusals and Medical Emergency calls, rather than from morning meetings.

The relationship between the surgeries planned that required an ICU bed and the elective surgeries cancelled because of the ICU being at full capacity was examined using a chi-square test of independence, a p-value <.05 was considered statistically significant.

To determine if there were baseline changes in ICU demand or workload over the study period, simple linear regression analyses were performed using data on HPPD, ICU bed occupancy, average Length of Stay (LoS) and monthly number planned elective surgeries. To account for the number of analyses in the linear regression analyses, an adjusted p-value <.0125 was considered statistically significant.

Qualitative method

Semi-structured interviews were held with ICU nurses, ICU doctors and hospital management five years post implementation. The interviews were conducted by ER, as part

For peer review only

of a research project for her studies at medical faculty after training by the RC-W and EA that are experienced in interviewing techniques. 12 staff members were approached, in person, by telephone or mail, by one of the research team and asked to participate. They were chosen by convenience, whilst ensuring that a mix of leaders and clinicians from the hospital and ICU were included. None of the participants knew the interviewer before the study, they were informed about the goal with the interviews and research project, and all consented to be interviewed. The number of interviews was based on the number of interviews needed for data saturation in the previous study.[6] The interview guide was designed to collect the staff member's perception of the plan and problems surrounding the implementation of the intervention (appendix A). The interviews were held at the interviewees' workplace with only the interviewer present. They were digitally recorded and professionally transcribed verbatim. Inductive interpretive analysis was performed to find key themes of the transcripts.[13] One researcher (ER) made the first analysis in which codes, subthemes and themes were developed. Codes were checked with two other researchers (RC-W and EA), who separately coded one of the interviews, and consensus was reached through discussion. A second researcher (AR) read all transcripts and verified the key themes from the analysis. Themes and sub-themes were validated through feedback and discussion with a hospital executive and ICU consultant doctor (PL), who has been familiar with the ICU and the intervention over its full implementation period.

Patient involvement

Patients or other members of the public were not involved in this study.

Results

Quantitative results

More planned elective surgeries requiring an ICU bed have been cancelled per year because of full capacity at the ICU, (X^2 (4, N=3299) =16.38, p=.003). Since 2015, there have been no changes in the number of beds in the ICU. The number of cancelled surgeries tripled between the year following the intervention (year 1) and year 2 (Table 1). The percentage of cancelled surgeries increased, beginning from one-year post-intervention, and has since maintained between 3.1% - 4.8%.

In the analysis of baseline changes in ICU demand or workload over the study period all variables were normally distributed except for average LoS, which was normally distributed

For peer review only

after logarithmic transformation. HPPD has increased over the years since 2014, ($F(1,1982)=35.99$, $p<.001$), with an R^2 of .018 (Figure 2a). For occupancy the regression equation was not significant ($F(1,1982)=5.21$, $p=.023$) (Figure 2b). The scatterplot of occupancy by date suggests that there is a discrepancy in how the data was recorded prior to mid-2016 as the data points are grouped around distinct values. There is however no clear pattern and should therefore not bias the results.

The data for both average LoS (Figure 2c) and number of planned surgeries requiring ICU beds (Figure 2d) indicates that a curvilinear relationship fits the model better than a linear relationship. Therefore, the linear model was transformed by including a square of the independent variable 'month' in the linear regression equation. This polynomical regression produces two regression coefficients in addition to the intercept. For average LoS the coefficient for the linear variable was positive and significant ($p=.001$), the coefficient on the quadratic variable was negative and significant ($p<.001$). This indicates that average LoS initially increased until a turning point after which it decreased over time $F(2,63)=6.76$, adjusted R^2 is .15. For number of planned surgeries requiring ICU beds, the coefficient for the linear variable was negative and significant ($p=.002$) while the coefficient on the quadratic variable was positive ($p<.001$) suggesting that the relationship is negative until a turningpoint after which the relationship is positive ($F(2,63)=11.79$, adjusted R^2 is .27).

Qualitative results

The multidisciplinary morning meetings implemented as part of the original intervention are still run at 8.00 o'clock every morning and the bed status is still recorded for the escalation plan in the meeting. 12 face to face interviews were conducted, four in each group – ICU doctors, ICU nurses and hospital managers. Interview length ranged between 18 to 46 minutes (average 30 minutes). All but two participants had previous knowledge about the 2014 implementation.

Themes

Data saturation was achieved since no new themes emerged from the later interviews. In the analysis, two themes with associated subthemes emerged: sustained benefits of implementation, and why implemented changes have not been sustained (Table 2). For complete code schedule see appendix B.

For peer review only

Sustained benefits of implementation

Positive experiences

In a busy environment such as an ICU, where many professions work together to solve problems, teamwork is vital. In the interviews, examples were given that the morning meeting supported teamwork: *"It would ensure that if there was things came up like the pharmacy, that the patient was going to be getting their medications at the right time because they were made available"* (manager 1). This also made the ICU *"... a nicer place to work"* (nurse 1).

Furthermore, the morning meeting was beneficial because it facilitated communication within the ICU as *"it is easy – effective transmission of knowledge*" (doctor 1) and is *"a useful forum of discussion"* (doctor 3). The meetings facilitated work by bringing attention to what was going to happen during the day. For example, *"... the pharmacist can start ordering specialist drugs if you need them ..."* (manager 1).

One benefit of the escalation plan was that it created a plan for how to operate when work was getting very hectic since "*... it's made us more aware of forward planning and being proactive"* (manager 1). One example was when patients were transferred to a neighbouring hospital in accordance with the plan, another is that it increased the ICU consultants' authority to say no to elective surgeries.

Enabling factors

That the morning meetings are the first activity of the day, and are held with all staff together, enabled its continuation. It was "*... the only point in time you can have everybody to sit down"* (manager 1). The morning meetings were beneficial from a human resources perspective because it ensured that *"people come to work on time"* (doctor 3). The visually displayed feedback of the colour coded bed status was thought to be beneficial. One participant reported that *"it's easier to do it [record bed status] because we are doing it in a handover meeting in the morning"* (doctor 2). The implementation worked well for a while because *"everybody was on the right page"* (nurse 1), and *"... it worked because it was something everyone was willing to try" (*doctor 4).

Why the implementation has not been sustained

Management of operations

Most participants reported that the pressure for elective surgeries has increased over the last five years and that there is a need for more resources being allocated for the ICU. For example, *"there's a massive push to meet our NEST [National Elective Surgery] targets and we are behind …"* (doctor 1). And *"If there's no more money for ICU then we just have to accept that there's going to be more cancellations and longer delays, that's reality"* (manager 3).

Some participants expressed that there were hierarchical barriers that created a negative workplace culture, *"…there are good leaders and there are bad leaders and there are good leaders that help people progress... Then there are some people that just put barriers in front of people …"* (nurse 2). All but two participants believed that the leadership do not understand the work done by frontline staff. One nurse expressed this as *"I think people are actually considering themselves a number"* (nurse 2).

Lack of communication and understanding

A lack of transparency about how nurses were rostered onto shifts was expressed, "*there has been loss of transparency about the staffing that we do have in the unit… and that actual discussion about where we are, has been lost"* (doctor 1). Furthermore, there was a discrepancy between how nurses and doctors view the number of available beds. The nurses thought they had the capacity to fill up the ICU with elective cases, by increasing the nurse staff with flex time, whereas the doctors wanted to keep beds free in anticipation of emergency cases. The communication problems also extended outside the ICU resulting in communication problems with other wards and up to management level. For example, *"They have different booking staff down there that don't really know what goes on up here "*(nurse 1*),* and *"…ICU need to speak the same language with the executive and surgical service group managers. We are not speaking the same language"* (doctor 2). It was also reported that some staff are unfamiliar with the escalation plan. Bed status was only visual at morning meetings and therefore *"there is no eyeballing of that"* (doctor 1) which makes it easier to forget. On occasion, the staff didn't understand the point of the escalation plan, "*So the numbers are actually useless. The colour code is useless. It's only for internal use."* (doctor 2).

For peer review only

Plans not working as intended

Over the five years since it was introduced, the use of the escalation plan has changed. There are several examples of when the staff member needs go the extra length in order to perform their duties *"I can't count the number of times I have brought my food to the bedside to eat"* (nurse 4). Some participants also expressed that the escalation plan wasn't comprehensive enough and that it should include a plan for how to respond to emergency admissions.

Bed status was not filled out in the afternoon as regularly as was intended *"because busy-ness, and the setting makes it easier to forget"* (doctor 2). Several examples were given when the traffic light system did not work as intended, "*it's called green when we should be amber, it's called amber when we should be red"* (doctor 3), and *"there was no major result from"* escalating it up to management level (manager 2).

High workload creates a negative workplace where *"Everyone's glum. There's no smiley. No happiness. No nothing and everything goes wrong."* (nurse 2), and *"... we couldn't bring the patient to ICU because there was no nurse to look after the patient. That actually made me go sick. ... I actually took time off sick, stress leave..."* (doctor 4).

Reality of clinical work

Lastly, there are factors that emerged over the intervening years that have affected the flow of patients that the escalation plan either didn't foresee or was unable to accommodate. For example, the introduction of electronic medical records, and delayed ward discharges. A common problem at ICUs is large variations in workload, and they are *"... either flooded or dry as, we never get that rhythm happening for any period of time"* (manager 2).

A common view was that the reason for the escalation plan not working was that there were not enough nurses and that "*they're not always available when you need them."* (doctor 4). A high staff turn-over rate with many newly recruited nurses was mentioned as detrimental for the work in the ICU "*we struggle, because we started to take graduates as well. They are only doubled up for a number of weeks and then we've got critically ill patients. That takes a lot more of the float nurse's time..."* (nurse 4).

For peer review only

Discussion

The intervention in 2014 aimed to reduce the number of cancelled elective surgeries due to the unavailability of ICU beds and to improve relations between the surgery department and the ICU. Even though the intervention in itself has continued to exist (i.e. the morning meeting and the escalation plan), the combined positive effects of the changes in the first year after implementation have not been sustained over time, with more elective surgeries cancelled due to the unavailability of ICU beds from two years post-intervention onwards. The main explanations for this were deficiencies in management of operations, lack of communication and understanding in the ICU and between ICU and other departments and hospital management, and other factors that the escalation plan and its implementation had not considered.

The current study is a five-year post-implementation follow up study which gives insight in the long-term effect after implementation of an intervention. It has been suggested that in order to investigate if changes have been sustained, the evaluation should be done at least one year after the intervention.[1, 14]

A major finding in the study is the lack of communication within ICU and between ICU and other departments, i.e. within the microsystem and between the micro- and mesosystems. Especially, discrepancies were found between how nurses and doctors saw availability of ICU beds. The microsystems that build complex adaptive systems such as healthcare have to continually adapt to changing conditions. Therefore, communication within the microsystem and between microsystems is important for the co-ordination of responses to changes and challenges for good adaptations; for risk management in complex systems, communication between different parts of the system is necessary.[15] A three-year follow-up study by Greenhalgh et al. found that good interrelations in the work environment are important for sustaining implementation.[16] They stated that when there is a lack of transparency with decision-making and workways, the communication is lost, and the microsystems cannot co-operate in harmony, which accords with our findings.

The multi-disciplinary meetings seem to have more positive effects associated with them than the escalation plan. That the morning meetings are always at the same time and in the same room is habit forming, which has been suggested to be an important element in sustaining change.[17] Several aspects of the meeting, such as the opportunity to review patients and

For peer review only

communicate plans, support daily work in the ICU. As noted in the original study, the morning meeting facilitates coordination of the workday for several different professionals at the same time, and reinforces teamwork.[6] These positive effects of the morning meetings improve the conditions within the ICU and could explain why they have become an integrated part of daily work.[18]

The increase of cancelled elective surgeries is not explained by a higher bed occupancy or increase in average length of stay at the ICU. However, planned elective surgeries has increased and HPPD has significantly increased. This indicates that both demand for ICU post-surgical beds and the workload for the nurses has increased and that the increased pressure for elective surgeries that the participants expressed in the interviews was indeed true. The increased pressure for elective surgery can create a patient safety problem if the workload pressure exceeds capacity at the ICU. When the workload and the management pressure increase, work in the organisation is pushed towards the margin of safe operations, and if this margin is passed the risk of patient harm increases.[19] To know this, and to work under that kind of pressure, may lead to problems with staff well-being, which was exemplified in the interviews.[20]

Even though most of the participants worked at the ICU during the implementation in 2014, there seems to have been a lack of understanding of why there is a need for the escalation plan. The original study found that there was no consistent agreement on the purpose of the plan,[6] and this lack of clear purpose could explain why commitment and dedication to the escalation plan has been lost over the years. For improvement leaders, be that management or engaged clinicians, it is important to be able to explain the purpose of the intervention, and to support knowledge and understanding among staff in organisations for long term good quality outcomes.[21] Furthermore, Staines et al. noted in a study of long-term success of quality work in a health care organisation the importance of leadership commitment to improvement work and support to front-line staff work with improvements.[22] Thus, continuous follow-up by management to reinforce commitment is needed for a change to be fully accepted and firmly established at the workplace.

Several participants thought that the escalation plan was not sufficiently comprehensive to cover all situations, and that the varied circumstances encountered in real clinical work was an obstacle for the implementation of the plan. However, when new processes are introduced

For peer review only

in complex adaptive systems, it can't be expected that they will stay the same since the conditions are always changing. It is therefore important to acknowledge the difference between Work-as-Imagined (WAI) and Work-as-Done (WAD).[23] It might be that the escalation plan, being WAI, is not flexible enough for the dynamics in the actual work going on in the ICU (i.e. WAD). This thinking accords with the interviews, where situations were reported when the escalation plan was not used as it should have been since bed status classification did not fit reality. The original study, however, highlighted how the plan was co-opted by staff members in different ways, according to their own needs, suggesting that the plan was sufficiently flexible when it was first implemented.[6] It is possible that some of that flexibility has been lost when some of the understanding and knowledge about the rationale for the escalation plan was lost. It is important that interventions are able to adapt to the changing context of healthcare to continuously refine and improve interventions to ensure they are able to be sustained.[24]

Study limitations

One limitation of the current study is that bed status data was incomplete from October 2016 to May 2018. This does not affect the most important findings since the study was done five years after implementation in 2019. Furthermore, in the regression analysis the R^2 is small for most analyses which suggests that the independent variable is only explaining a part of the variance in the outcome variable. Thus, it is possible that there are other factors that might also explain the variance in the number of cancelled surgeries for example staff and policies changes or baseline increase in demand due to aging population. These factors could not be evaluated within the scope of this study.

Conclusion

In this study, the interventions have been partly sustained over time but the positive effects of have not. Thus, there is no such thing as a 'fix and forget' solution for implementation of interventions in complex adaptive systems as healthcare. Management commitment to support communication within and between microsystems, and to support health care staff understanding of the underlying reasons for intervention are necessary for success over time.

For peer review only

Author contribution

All authors contributed to the planning and design of the study; ER collected the study data, and made the first analysis and interpretation, which were then finalised together with AR, RC-W and EA. ER and AR drafted the manuscript; all authors revised it critically for intellectual content and approved the final version of the paper.

Funding

This research received no specific grant from any funding agency in the public, commercial or not-for profit sectors.

Competing interest

PL and AJ were involved in development of the intervention. They were however not interviewed as part of the study and were not involved in data collection or analysis. The authors have no other competing interests to declare.

Patient consent form

Not required.

Ethics approval

The Human Research Ethics Committee at the studied hospital (HREC/2019/QTHS/59796).

Data sharing statement

No data are available except those uploaded as supplementary information. Study data cannot be obtained by a third party as a requirement of ethics approval.

References

1. Scheirer MA, Dearing JW. An agenda for research on the sustainability of public health programs. *Am J Public Health.* 2011;101(11):2059-2067. doi: 10.2105/AJPH.2011.300193
2. Mesner SA, Foster NE, French SD. Implementation interventions to improve the management of non-specific low back pain: a systematic review. *BMC Musculoskelet Disord*. 2016;17:258. doi:10.1186/s12891-016-1110-z [published 10 Jun 2016]

For peer review only

3. Buchanan D, Fitzgerald L, Ketley D, et al. No Going Back: A Review of the Literature on Sustaining Organizational Change. *International Journal of Management Reviews* 2005;7(3):189 - 205. doi: 10.1111/j.1468-2370.2005.00111.x.
4. Fixsen DL, Naoom SF, Blase KA, et al. (2005). Implementation research: A synthesis of the literature. Tampa, FL: University of South Florida, Louis de la Parte Florida Mental Health Institute, The National Implementation Research Network (FMHI Publication #231).
5. Martin GP, Weaver S, Currie G, et al. Innovation sustainability in challenging health-care contexts: embedding clinically led change in routine practice. *Health Serv Manage Res.* 2012;25(4):190–199. doi:10.1177/0951484812474246
6. Clay-Williams R, Blakely B, Lane P, et al. Improving decision making in acute healthcare through implantation of an intensive care unit (ICU) intervention in Australia: a multimethod study. *BMJ Open* 2019;9:e025041. doi:10.1136/bmjopen-2018-025041.
7. Braithwaite J, Clay-Williams R, Nugus P, et al. Health care as a complex adaptive system. In Hollnagel E, Braithwaite J, Wears RL, eds. Resilient Health Care. Farnham, Surrey, England: Ashgate Publishing. 2013: 57-73.
8. Nelson EC, Godfrey MM, Batalden PB, et al. Clinical Microsystems, Part 1. The Building Blocks of Health Systems. *Jt Comm J Qual Patient Saf.* 2008;34(7):367-78. doi: 10.1016/S1553-7250(08)34047-1
9. Mohr JJ, Batalden P, Barach P. Integrating patient safety into the clinical microsystem. *Qual Saf Health Care.* 2004;13:ii34–ii38. doi: 10.1136/qshc.2003.009571
10. Johnson JK, Batalden PB. Educating health professionals to improve care within the clinical microsystem. McLaughlin and Kaluzny's Continuous Quality Improvement In Health Care. Burlington: Jones & Bartlett Learning; 2013.
11. Scheirer MA. Is sustainability possible? A review and commentary on empirical studies of program sustainability. *Am J Eval.* 2005;26(3):320–347. doi:10.1177/1098214005278752
12. Kirby KK. Hours per Patient Day: Not the Problem, Nor the Solution. *Nurse Econ.* 2015;33(1):64-6.
13. Denzin NK, Lincoln YS. Strategies of qualitative inquiry. 4th edn. US. Sage, 2013.

For peer review only

14. Wiltsey Stirman S, Kimberly J, Cook N, et al. The sustainability of new programs and innovations: a review of the empirical literature and recommendations for future research. *Implement Sci.* 2012;7:17. doi:10.1186/1748-5908-7-17
15. Rasmussen J. Risk management in a dynamic society: a modelling problem. *Saf Sci.*1997;27(2-3):183-213. doi:10.1016/S0925-7535(97)00052-0
16. Greenhalgh T, Macfarlane F, Barton-Sweeney C, et al. "If we build it, will it stay?" A case study of the sustainability of whole-system change in London. *Milbank Q.* 2012;90(3):516–547. doi:10.1111/j.1468-0009.2012.00673.x
17. Fleiszer AR, Semenic SE, Ritchie JA, et al. An organizational perspective on the long-term sustainability of a nursing best practice guidelines program: a case study. *BMC Health Serv Res.* 2015;15:535. doi:10.1186/s12913-015-1192-6
18. Aston J, Shi E, Bullôt H, Galway R, Crisp J. Qualitative evaluation of regular morning meetings aimed at improving interdisciplinary communication and patient outcomes. *Int J Nurs Pract.* 2005;11(5):206-13. doi:10.1111/j.1440-172X.2005.00524.x
19. Cook R, Rasmussen J. "Going solid": a model of system dynamics and consequences for patient safety. *Qual Saf Health Care.* 2005;14(2):130–134. doi:10.1136/qshc.2003.009530
20. Endacott R. The continuing imperative to measure workload in ICU: impact on patient safety and staff well-being. *Intensive Care Med*. 2012;38(9):1415-1417. doi:10.1007/s00134-012-2654-5
21. Sandberg J, Targama A. Managing understanding in organizations. London: SAGE; 2007.
22. Staines A, Thor J, Robert G. Sustaining improvement? The 20-year Jönköping quality improvement program revisited. *Qual Manag Health Care.* 2015;24(1):21–37. doi:10.1097/QMH.0000000000000048
23. Clay-Williams R, Hounsgaard J, Hollnagel E. Where the rubber meets the road: using FRAM to align work-as-imagined with work-as-done when implementing clinical guidelines. *Implement Sci*. 2015;10:125. doi:10.1186/s13012-015-0317-y
24. Chambers DA, Glasgow RE, Stange KC. The dynamic sustainability framework: addressing the paradox of sustainment amid ongoing change. *Implementation Sci.* 2013;8:117. doi:10.1186/1748-5908-8-117

For peer review only

Table 1: Surgeries with post-operative ICU recovery, planned and cancelled

	Planned elective surgeries requiring an ICU bed, n	Cancelled surgeries due unavailability of an ICU bed, n (%)
Intervention year (Dec 2014 – Nov 2015)	681	8 (1.17)
Year 1 (Dec 2015 – Nov 2016)	580	18 (3.10)
Year 2 (Dec 2016 – Nov 2017)	560	27 (4.82)
Year 3 (Dec 2017 – Nov 2018)	736	27 (3.67)
Year 4 (Dec 2018 – Nov 2019)	742	33 (4.45)

Table 2: Themes and subthemes found in the analysis in the interviews

Themes	Sub-themes
Sustained benefits of implementation	Positive experiences
	Enabling factors
Why the implementation has not been sustained	Management of operations
	Lack of communication and understanding
	Plans not working as intended
	Reality of clinical work

For peer review only

Figure 1: The ICU escalation plan

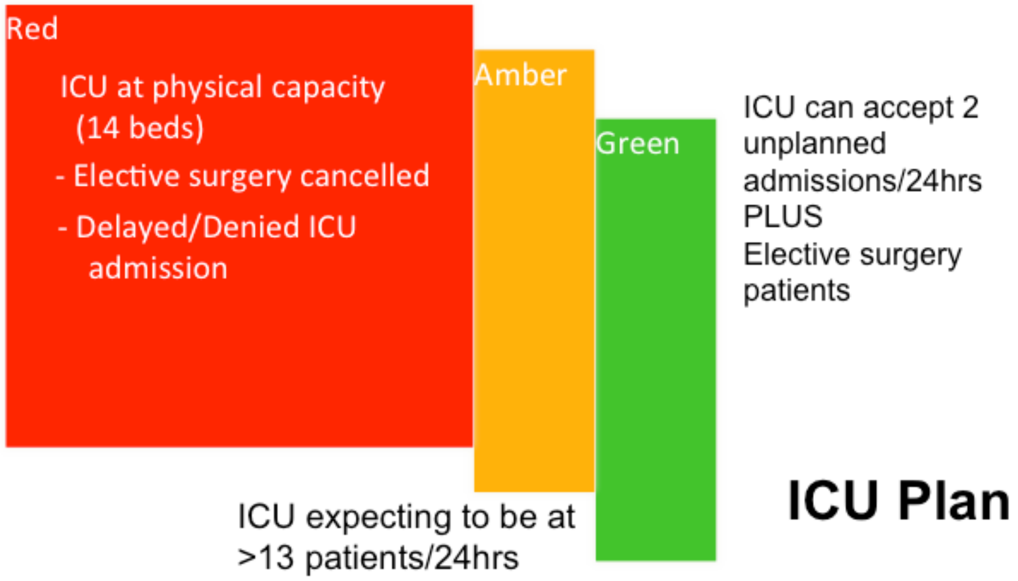

For peer review only

Figure 2: Changes in ICU demand or workload over the study period.

2a) Hours per patient day (HPPD) a measure of workload for nurses, per date.

2b) Occupancy at ICU, per date.

2c) Average Length of stay at ICU (LoS), per month.

2d) Monthly number of planned surgical admissions to ICU, per month.

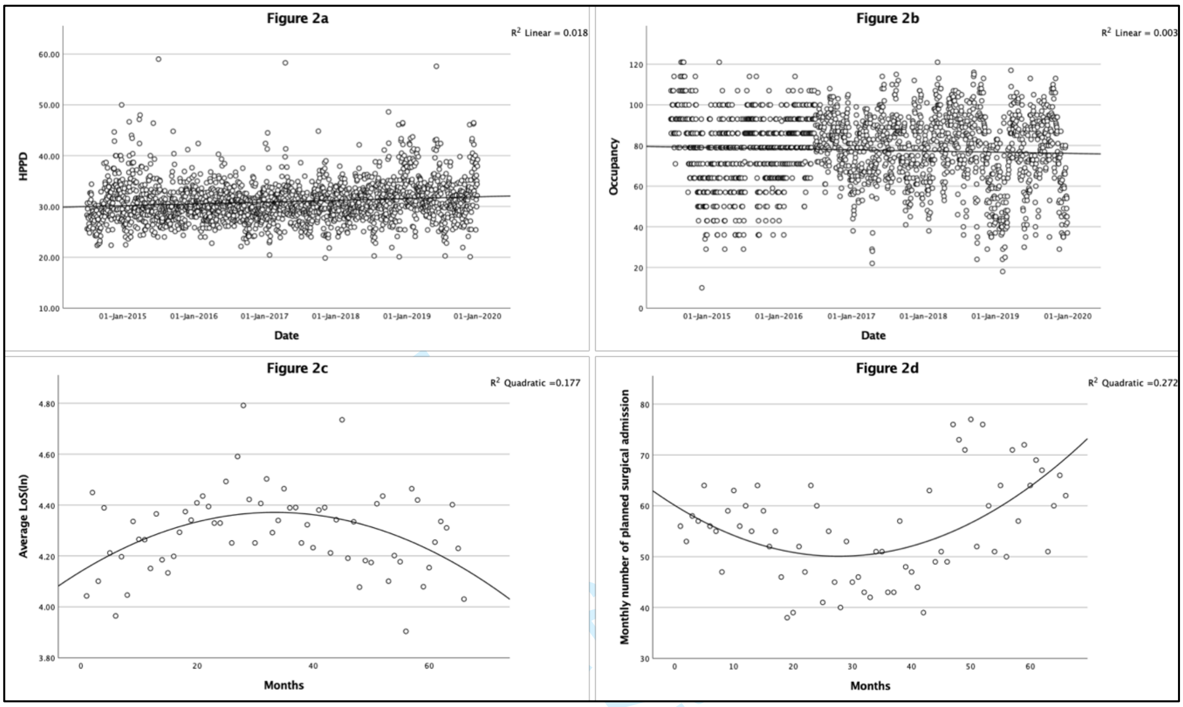

Appendix A: Interview Schedule

The information collected will be demographic information and responses to the following questions.

A: The ICU Escalation plan and morning handover meeting implementation

Do you know of the ICU Escalation plan and morning handover meetings that was implemented 2014?
Were you involved in the implementation process?
If so – what was your role?
What is your experience of the morning handover meetings (if relevant)?
What is your experience of the ICU Escalation plan (if relevant)?
Can you describe an episode when the ICU Escalation plan was activated (if relevant)?

B: ICU Resource allocation processes

Please describe how beds and resources are allocated in the ICU.
What positions have an input into these decisions?
What are the factors involved?
Does the process change as the ICU becomes busier?

C: Work practice changes

Questions to the hospital management group.
How does your work at the hospital involve the ICU?
Tell me how the ICU impacts on your daily work routine.
Are you aware of any change to your work practice due to the Escalation plan implementation?
Are you aware of any change to the work practices of other staff as a result of the initiatives?

Questions to ICU staff.
Are you aware of any change to your work practice due to the morning handover meetings?
Are you aware of any change to your work practice due to the Escalation plan implementation?
Are you aware of any change to the work practices of other staff as a result of the initiatives?

D: Job satisfaction

How do you feel about coming to work each day, particularly on days with heavy ICU involvement?
Was there any impact of the initiatives on your job satisfaction?
Did the initiatives make any difference to the days when ICU resources where stretched?
Do you feel patient care was improved?

For peer review only

E: Questions on the sustainability of the ICU Escalation Plan and daily morning meeting. Two different sets of questions to be used depending on the results of the analysis of the quantitative data.

I) *If the analysis of data shows that the improvement has sustained over time*
To your opinion – why has the benefits of the invention sustained over time?
What has been the key factors?
Who has been the key players? What did they do to help the success?
What suggestions do you have for the work with optimizing flow of patients through ICU?

II) *If the analysis of data shows that the improvement has <u>not</u> sustained over time*
To your opinion – why has the benefits of the invention not sustained over time?
What has been the obstacles?
What could have been done differently?
What suggestions do you have for the work with optimizing flow of patients through ICU?

F: Demographic Information

This information will not be linked to participants name. A number will be given to be used as a code reference for analysis purposes.

1.Gender
2.Profession
3.Role at TTHS
4.Time since qualification to practice his profession
5.Time in this organisation
6.Time in ICU

For peer review only

Appendix B: Code schedule

**Theme**	**Sub-theme**	**Code**	**Quotes**
Sustained benefits of implementation	Positive experiences	Facilitates communication within microsystem	Nurse 3: *"[…] as soon as you might say to one of the intensivists, where are we, and you can say, red, they know exactly the situation we're in. So, it's a good, quick identification."*
		Work-as-imagined reconciles work-as-done	Nurse 2: *"The doctors have been really good, so then they won't admit or […] they wouldn't admit high dependency patients. […] They've been very good supporting the staff and they've been - at one stage I remember them actually having to transfer to The Mater or something patients that had come into ED (emergency department), because we couldn't take them here, because he had no beds."*
		Improves teamwork	Manager 1: *"I think the morning meetings have made everybody more aware and tried to collaborate as a team approach, and I think that's really important."*
	Enabling factors	Morning meeting setting benefits	Manager 1: *"[…] it is continuous but that is the only point in time you can have everybody to sit down."*
		Everyone was invested	Doctor 4: *"Well, it worked because it was something everyone was willing to try."*
Why the implementation has been not sustained	Management of operations	Leadership not understanding micro level work	Doctor 3: *"I'm going to be harsh here, a lot of the senior nursing staff who are going to be making those decisions are not there at ten o'clock at night or two in the morning, having to actually wear the brunt, the consequences of those decisions."*

For peer review only

		Hierarchical barriers to improvement	Nurse 2: *"[...] sometimes there are good leaders and there are bad leaders and there are good leaders that help people progress and things like that. Then there are some people that just put barriers in front of people. There are some people that just like arguing for the sake of arguing, which doesn't do the unit very good."*
		Pressure for elective surgeries	Doctor 1: *"There's a massive push to meet our NEST targets and we are behind and our service group is, I think, the only one who ended up in the red financially because we didn't meet our targets."*
		Lack of ICU resources	Manager 3: *"[...] if we have the same amount of ICU beds next year, the number of elective surgeries we cancel will just increase. If there's no more money for ICU then we just have to accept that there's going to be more cancellations and longer delays, that's reality."*
	Lack of communication and understanding	Lack of knowledge about implementation	Nurse 3: *"I don't know whether the new registrars and the new shift coordinators have a full grasp of the traffic light system."*
		Micro doesn't understand the escalation plan	Doctor 2: *"At the borderline state it doesn't do a lot. When it's really severe, when reach beyond red, which is very rare, it could be useful."*
		Lack of communication and discrepancy within microsystem	Doctor 1: *"[...] there has been loss of transparency about the staffing that we do have in the unit. We are often told oh, don't worry about that or we'll get staff, or you don't need to know that or yep, so I think that the lack of transparency and that actual*

For peer review only

			discussion about where we are, has been lost.”
		Lack of communication and discrepancy between microsystems	Nurse 1: *“They have different booking staff down there that don’t really know what goes on up here and so they’ll just have a surgeon that wants to operate. He’s got a theatre, so they’ll say yes to anything not really realising that those patients do need to come to an intensive care unit […].”*
		Lack of communication between micro and meso	Doctor 2: *“[…] ICU need to speak the same language with the executive and surgical service group managers. We are not speaking the same language.”*
	Plans not working as intended	Misuse of traffic light	Manager 2: *“There's a lot of disagreement, I think, around even though we've all agreed on the criteria for red, there's still, on the day of, flexibility in the thinking that yes you can get that heart done or yes you can get that elective surgery case in or no you can't, that sort of give and take.”*
		Use of escalation plan has changed over time	Doctor 3: *“The process at the beginning was fine. It’s been progressive reinterpretation that’s given it grief.”*
		Extra effort needed	Nurse 4: *“There are many days where we haven't get breaks. We just don't get breaks. We can't get staff to the bedside to help with turns […]. You should have two nurses to turn them. You don't do that. You can't get them. People get cranky with each other because they're not getting their breaks. We get around things by - often - I can't count the number of times I have brought my food to the bedside to eat[…].”*

For peer review only

		Consequences of over-loaded ICU	Doctor 4: *"[...] we couldn't bring the patient to ICU because there was no nurse to look after the patient. That actually made me go sick. [...]I actually took time off sick, stress leave, and developed shingles, reactivation of chickenpox due to the stress."*
		Implementation not comprehensive enough	Manager 2: *"[...] it's very difficult to draft for everything that could happen and it's not black and white"*
	Reality of clinical work	Disturbance in patient flow	Manager 4: *"And then there's days where there's, potentially, patients who could go to the ward, and there's nowhere to send them to."*
		Increased workload for nurses	Nurse 2: *"I think the workload's increased for the nurses."*
		Staffing problem	Nurse 2: *"We have more new nurses. [...] For transition. Like, they've just gone to uni. They've had no ward - they've had no experience whatsoever and they're coming up here, so that takes a lot of time away."*
		New clinical and administrative processes	Doctor 2: *"So it's actually worse in the last year with the EMR (electronic medical record) because it increased our workload."*
		Natural variation	Doctor 1: *"[...] January and February are classically - and I think it happens every year, really slow months. Then around August is always a peak. It's around flu season and more people have MIs and things like that but it seems to be a - not*

For peer review only

			necessarily predictable but a constant variation."

For peer review only

COREQ (COnsolidated criteria for REporting Qualitative research) Checklist

A checklist of items that should be included in reports of qualitative research. You must report the page number in your manuscript where you consider each of the items listed in this checklist. If you have not included this information, either revise your manuscript accordingly before submitting or note N/A.

Topic	Item No.	Guide Questions/Description	Reported on Page No.
**Domain 1: Research team and reflexivity**			
Personal characteristics			
Interviewer/facilitator	1	Which author/s conducted the interview or focus group?	6
Credentials	2	What were the researcher's credentials? E.g. PhD, MD	6
Occupation	3	What was their occupation at the time of the study?	6
Gender	4	Was the researcher male or female?	6
Experience and training	5	What experience or training did the researcher have?	6
Relationship with Participants:			
Relationship established	6	Was a relationship established prior to study commencement?	6
Participant knowledge of the interviewer	7	What did the participants know about the researcher? e.g. personal goals, reasons for doing the research	6
Interviewer characteristics	8	What characteristics were reported about the inter viewer/facilitator? e.g. Bias, assumptions, reasons and interests in the research topic	6
**Domain 2: Study design**			
Theoretical framework			
Methodological orientation and Theory	9	What methodological orientation was stated to underpin the study? e.g. grounded theory, discourse analysis, ethnography, phenomenology, content analysis	6
Participant selection			
Sampling	10	How were participants selected? e.g. purposive, convenience, consecutive, snowball	6
Method of approach	11	How were participants approached? e.g. face-to-face, telephone, mail, email	6
Sample size	12	How many participants were in the study?	6
Non-participation	13	How many people refused to participate or dropped out? Reasons?	N/A
Setting			
Setting of data collection	14	Where was the data collected? e.g. home, clinic, workplace	6
Presence of non-participants	15	Was anyone else present besides the participants and researchers?	6
Description of sample	16	What are the important characteristics of the sample? e.g. demographic data, date	7
Data collection			
Interview guide	17	Were questions, prompts, guides provided by the authors? Was it pilot tested?	6
Repeat interviews	18	Were repeat inter views carried out? If yes, how many?	N/A
Audio/visual recording	19	Did the research use audio or visual recording to collect the data?	6
Field notes	20	Were field notes made during and/or after the inter view or focus group?	N/A
Duration	21	What was the duration of the inter views or focus group?	7
Data saturation	22	Was data saturation discussed?	8
Transcripts returned	23	Were transcripts returned to participants for comment and/or correction?	N/A

For peer review only

Topic	Item No.	Guide Questions/Description	Reported on Page No.
**Domain 3: analysis and findings**			
Data analysis			
Number of data coders	24	How many data coders coded the data?	6
Description of the coding tree	25	Did authors provide a description of the coding tree?	8
Derivation of themes	26	Were themes identified in advance or derived from the data?	6, 8
Software	27	What software, if applicable, was used to manage the data?	N/A
Participant checking	28	Did participants provide feedback on the findings?	6
Reporting			
Quotations presented	29	Were participant quotations presented to illustrate the themes/findings? Was each quotation identified? e.g. participant number	8-11
Data and findings consistent	30	Was there consistency between the data presented and the findings?	Yes
Clarity of major themes	31	Were major themes clearly presented in the findings?	8-11
Clarity of minor themes	32	Is there a description of diverse cases or discussion of minor themes?	8-11

Developed from: Tong A, Sainsbury P, Craig J. Consolidated criteria for reporting qualitative research (COREQ): a 32-item checklist for interviews and focus groups. *International Journal for Quality in Health Care*. 2007. Volume 19, Number 6: pp. 349 – 357

For peer review only

BMJ Open

Sustainment of a patient flow intervention in an Intensive Care Unit in a regional hospital in Australia: a mixed-method, five-year follow-up study

Journal:	*BMJ Open*
Manuscript ID	bmjopen-2020-047394.R1
Article Type:	Original research
Date Submitted by the Author:	17-May-2021
Complete List of Authors:	Ros, Eva; Linköping University, Department of Biomedical and Clinical Sciences; Macquarie University Australian Institute of Health Innovation<br>Ros, Axel; Jönköping University, Jönköping Academy for Improvement of Health and Welfare, The School of Health and Welfare, ; Macquarie University Australian Institute of Health Innovation<br>Austin, Elizabeth; Macquarie University, Australian Institute of Health Innovation<br>De Geer, Lina; Linköping University Hospital, Department of Anaesthesiology and Intensive Care ; Linköping University, Department of Biomedical and Clinical Sciences<br>Lane, Paul; Townsville Hospital and Health Service<br>Johnson, Andrew; Townsville Hospital and Health Service; James Cook University College of Medicine and Dentistry<br>Clay-Williams, Robyn; Macquarie University, Australian Institute of Health Innovation
**Primary Subject Heading**:	Medical management
Secondary Subject Heading:	Qualitative research
Keywords:	Health & safety < HEALTH SERVICES ADMINISTRATION & MANAGEMENT, Quality in health care < HEALTH SERVICES ADMINISTRATION & MANAGEMENT, Adult intensive & critical care < INTENSIVE & CRITICAL CARE, QUALITATIVE RESEARCH

SCHOLARONE™
Manuscripts

I, the Submitting Author has the right to grant and does grant on behalf of all authors of the Work (as defined in the below author licence), an exclusive licence and/or a non-exclusive licence for contributions from authors who are: i) UK Crown employees; ii) where BMJ has agreed a CC-BY licence shall apply, and/or iii) in accordance with the terms applicable for US Federal Government officers or employees acting as part of their official duties; on a worldwide, perpetual, irrevocable, royalty-free basis to BMJ Publishing Group Ltd ("BMJ") its licensees and where the relevant Journal is co-owned by BMJ to the co-owners of the Journal, to publish the Work in this journal and any other BMJ products and to exploit all rights, as set out in our licence.

The Submitting Author accepts and understands that any supply made under these terms is made by BMJ to the Submitting Author unless you are acting as an employee on behalf of your employer or a postgraduate student of an affiliated institution which is paying any applicable article publishing charge ("APC") for Open Access articles. Where the Submitting Author wishes to make the Work available on an Open Access basis (and intends to pay the relevant APC), the terms of reuse of such Open Access shall be governed by a Creative Commons licence – details of these licences and which Creative Commons licence will apply to this Work are set out in our licence referred to above.

Other than as permitted in any relevant BMJ Author's Self Archiving Policies, I confirm this Work has not been accepted for publication elsewhere, is not being considered for publication elsewhere and does not duplicate material already published. I confirm all authors consent to publication of this Work and authorise the granting of this licence.

For peer review only

Sustainment of a patient flow intervention in an Intensive Care Unit in a regional hospital in Australia: a mixed-method, five-year follow-up study

Author Eva Ros 1) 2)
Co-authors Axel Ros 3) 2), Elizabeth E. Austin 2), Lina De Geer 4) 1), Paul Lane 5), Andrew Johnson 5) 6), Robyn Clay-Williams 2).

1) Department of Biomedical and Clinical Sciences, Linköping University, Linköping, Sweden

2) Australian Institute of Health Innovation, Macquarie University, Sydney, Australia

3) Jönköping Academy for Improvement of Health and Welfare, The School of Health and Welfare, Jonkoping University, Jonkoping, Sweden

4) Department of Anaesthesiology and Intensive Care in Linköping, Sweden

5) Townsville Hospital and Health Service, Townsville, Australia

6) College of Medicine and Dentistry, James Cook University, Townsville, Australia

Corresponding author:
Eva Ros
Vattengatan 36
60220 Norrkoping
Sweden

E-mail: evaro626@student.liu.se
Phone: +4673-525 17 41
Fax number: No fax number available

Keywords: implementation science, change management, mixed-method study, quality of health care, intensive care units

Word count: 4024

For peer review only

Abstract

Objective

In 2014 an escalation plan and morning handover meetings were implemented in an Intensive Care Unit (ICU) to reduce access block for post-operative care. In this study, the improvement intervention is revisited five years on with the objective to see if the changes are sustained and to understand factors contributing to sustainability.

Design

A mixed-method approach was used, with quantitative analysis of ICU administrative data and qualitative analysis of interviews with hospital management and ICU staff.

Setting

ICU with mixed surgical and non-surgical cases in a regional hospital in Australia.

Participants

Interview participants: ICU nurses (four), ICU doctors (four) and hospital management (four).

Main outcome measures

Monthly number of elective surgeries cancelled due to unavailability of ICU beds. Staff perceptions of the interventions and factors contributing to sustainability.

Results

After a decline in elective surgeries being cancelled in the first year after the intervention, there was an increase in cancellations in the following years (X^2=16.38, p=.003). Lack of knowledge about the intervention and competitive interests in the management of patient flow were believed to be obstacles for sustained effects of the original intervention. So were communication deficiencies that were reported within the ICU and between ICU and other departments. There are discrepancies between how nurses and doctors use the escalation plan and regard the availability of ICU beds.

Conclusion

Improvement interventions in healthcare that appear initially to be successful are not necessarily sustained over time, as was the case in this study. In healthcare, there is no such thing as a 'fix and forget' solution for interventions. Management commitment to support communication within and between microsystems, and to support healthcare staff understanding of the underlying reasons for intervention, are important implications for change and change management across healthcare systems.

For peer review only

Strength and limitations of this study

- This is a five-year follow-up study of an implementation of an intervention.
- A mixed-method approach with both quantitative and qualitative analysis provides an important insight to both understand if the implementations had sustained or not and describe why it had or had not sustained.
- The mixed-method approach used also made it possible to guide the interviews from the quantitative results, but also to verify some of what has been stated in interviews with the quantitative results.
- The statistical analysis suggests that there might be other factors that could affect the rate of cancelled surgeries such as staff and policies changes, or baseline increase in demand due to for example an aging population.

Background

The long-term sustainability of interventions implemented in healthcare is important, but seldom evaluated. More typically, the effectiveness of an intervention is assessed shortly after implementation and then the evaluation is deemed to be complete.[1] Single interventions or one-off implementation of an intervention bundle may seem attractive, but they are largely unsuccessful in effecting meaningful change over time in clinical practice.[2] The definition of sustainability is somewhat controversial.[1, 3] It can be described as something steady that does not revert, but also as a dynamic state where the intervention can adapt to new situations.[4, 5] From a quality and patient safety viewpoint, the importance of an intervention should be the effect of it, not the intervention itself.

Bed occupancy at Intensive Care Units (ICU) can show unexpected large variations due to admission of critical patients. It is therefore a challenge to balance the capacity for planned elective surgeries requiring intensive care after surgery with the unplanned critical patients in need of intensive care, particularly in hospitals with a single ICU for both categories of patients. In 2014, senior clinical staff at the ICU at a regional hospital in Australia saw a need to optimise the patient flow through the ICU to decrease cancellations of elective surgeries due to the unavailability of ICU beds.[6] An ICU escalation plan, a daily multidisciplinary morning handover meeting and optional education in system resilience for staff members were developed and implemented at the end of November 2014. The escalation plan, based on three different states – green, amber and red, was introduced as a system to indicate bed

For peer review only

availability. After a follow-up period of ten months the intervention was found to have succeeded, since patient flow improved and elective surgeries cancelled due to unavailable ICU beds, the key performance indicator of the intervention, was significantly reduced.[6] Improved internal communication, decision-making and cohesion within the ICU and better coordination between ICU and other hospital departments, such as surgery and the emergency department, were found to contribute to the success of the intervention.

Improvements in healthcare must be sustainable. Yet we know that healthcare practices often revert to previous practice once the energy and funding associated with the original intervention are removed.[1] Hence, it is critical to study the long-term sustainability of interventions in addition to the immediate outcomes. A review of evidence-based studies of intervention sustainability found that only 40-60% of interventions are sustained over time.[7] Most of the studies focused on the sustainability of activities, only a few on performance indicator outcomes. In research on sustainability of interventions it is important to study both the sustainment of the outcome of the intervention, and of the practices.[1] It has been suggested that researcher site visits to interview multiple informants are valuable to study the sustainment of practices.[1]

The current study revisited the implementation of an ICU escalation plan and morning meeting at the same regional hospital five years on, with the objectives:

(1) To find if the effect of the 2014 implementation has been sustained over time or not, and
(2) To understand why the effect of implementation has been sustained or not.

The setting is a fourteen bed ICU with mixed surgical and non-surgical cases. There have been no changes in the number of beds, no significant changes in staffing, and no other patient flow interventions, in the ICU during the studied period.

Method

We conducted a mixed-method study. First, quantitative data were collected to examine the effect of the intervention over time. Second, qualitative data on staff perspectives regarding the intervention's sustainability was collected.

Quantitative method

ICU audit data was collected from July 2014 to December 2019. The monthly number of cancelled surgeries due to no available ICU beds was collected to understand if the initial

For peer review only

implementation had sustained or not. The monthly number of planned elective surgeries demanding ICU beds, nursing Hours Per Patient Day (HPPD; a measure of workload for nurses),[8] bed occupancy and length of stay (LoS) at the ICU was collected to decide if they influenced cancellation rate. Bed status (green, amber, red) was determined from notes recorded at the ICU morning meetings and from referrals, refusals and Medical Emergency calls to assess the compliance to bed status registration.

The relationship between the surgeries planned that required an ICU bed and the elective surgeries cancelled because of the ICU being at full capacity was examined using a chi-square test of independence, a p-value <.05 was considered statistically significant.

A p-chart was calculated based on monthly numbers of cancellations to show the trend of cancellations. Since too many months had zero cancellations to allow for the analyses to be made by month, quarters (three consecutive months) were used as the base for the analyses, starting from the first month after the intervention.

To determine if there were baseline changes in ICU demand or workload over the study period that could influence the result of the key performance indicator, cancellations of surgery, simple linear regression analyses were performed using data on HPPD, ICU bed occupancy, average LoS and monthly number planned elective surgeries. To account for the number of analyses in the linear regression analyses, an adjusted p-value <.0125 was considered statistically significant.

Descriptive statistics (number, percentage) were used to tabulate results. SPSS (v27) and SPC XL 2020 (v2.50.0600) was used for statistical analysis.

Qualitative method

Semi-structured interviews were held with ICU nurses, ICU doctors and hospital management five years post-implementation. The interviews were conducted by ER, as part of a research project for her studies at medical faculty after training by RC-W and EA that are experienced in interviewing techniques. The staff members were approached, in person, by telephone or mail, by one of the research team and asked to participate. They were chosen by convenience, whilst ensuring that a mix of leaders and clinicians from the hospital and ICU

For peer review only

were included. None of the participants knew the interviewer before the study, they were informed about the goal with the interviews and research project, and all consented to be interviewed. All but two participants had previous knowledge about the 2014 implementation. The number of interviews was based on the number of interviews needed for data saturation in the previous study.[6] The interview guide was informed by the result of the quantitative analysis and designed to collect the staff member's perception of the plan and problems surrounding the implementation of the intervention (appendix A). The interviews were held at the interviewees' workplace with only the interviewer present. They were digitally recorded and professionally transcribed verbatim. Inductive interpretive analysis was performed to find key themes of the transcripts.[9] One researcher (ER) made the first analysis in which codes, subthemes and themes were developed. Codes were checked with two other researchers (RC-W and EA), who separately coded one of the interviews, and consensus was reached through discussion. A second researcher (AR) read all transcripts and verified the key themes from the analysis. Themes and sub-themes were validated through feedback and discussion with a hospital executive and ICU consultant doctor (PL), who is familiar with the ICU and the intervention over its full implementation period.

Patient involvement

Patients or other members of the public were not involved in this study.

Results

Quantitative results

The mean cancellation rate was 6.9% in the five months preceding the intervention. After a decline in elective surgeries being cancelled per year in the first year after the intervention, there was an increase in cancellations in the following years (X^2 (4, N=3299) =16.38, p=.003). The percentage of cancelled surgeries per year increased, beginning from one year post-intervention, and has since maintained between 3.1% - 4.8%. The number of cancelled surgeries tripled in the years following the first year after the intervention (Table 1). The trend of the cancellation rate is shown in a control chart in Figure 1. The high variability around the mean of cancellations in the period after the initial improvement implies that the process of cancellations of surgery is instable.

In the analysis of baseline changes in ICU demand or workload over the study period, all variables were normally distributed except for average LoS, which was normally distributed

For peer review only

after logarithmic transformation. HPPD has increased over the years since 2014, ($F(1,1982)=35.99$, $p<.001$), with an R^2 of .018 (Figure 2a). For occupancy, the regression equation was not significant ($F(1,1982)=5.21$, $p=.023$) (Figure 2b). The scatterplot of occupancy by date suggests that there is a discrepancy in how the data was recorded prior to mid-2016 as the data points are grouped around distinct values. There is however no clear pattern and should therefore not bias the results.

The data for both average LoS (Figure 2c) and the number of planned surgeries requiring ICU beds (Figure 2d) indicates that a curvilinear relationship fits the model better than a linear relationship. Therefore, the linear model was transformed by including a square of the independent variable 'month' in the linear regression equation. This polynomial regression produces two regression coefficients in addition to the intercept. For average LoS the coefficient for the linear variable was positive and significant ($p=.001$), the coefficient on the quadratic variable was negative and significant ($p<.001$). This indicates that average LoS initially increased until a turning point after which it decreased over time $F(2,63)=6.76$, adjusted R^2 is .15. For the number of planned surgeries requiring ICU beds, the coefficient for the linear variable was negative and significant ($p=.002$) while the coefficient on the quadratic variable was positive ($p<.001$) suggesting that the relationship is negative until a turning point after which the relationship is positive ($F(2,63)=11.79$, adjusted R^2 is .27).

Daily bed status registration was observed in notes recorded at the ICU morning meetings and from referrals, refusals and Medical Emergency calls. However, the data was incomplete from October 2016 to May 2018 and completely missing from July 2017 to February 2018. According to hospital officials, bed status was registered in that period, however registration data are lost.

Qualitative results

The multidisciplinary morning meetings implemented as part of the original intervention are still running at 8.00 o'clock every morning and the bed status is still recorded for the escalation plan in the meeting. Twelve face to face interviews were conducted, four in each group – ICU doctors, ICU nurses and hospital managers. Interview length ranged between 18 to 46 minutes (average 30 minutes).

For peer review only

Themes

Data saturation was achieved since no new themes emerged from the later interviews. In the analysis, two themes with associated subthemes emerged: sustained benefits of implementation, and factors adversely affecting sustainability (Table 2). For the complete coding schedule see appendix B.

Sustained benefits of implementation

Positive experiences

In a busy environment such as an ICU, where many professions work together to solve problems, teamwork is vital. In the interviews, examples were given that the morning meeting supported teamwork: *"It would ensure that if there was things came up like the pharmacy, that the patient was going to be getting their medications at the right time because they were made available"* (manager 1). This also made the ICU *"… a nicer place to work"* (nurse 1).

Furthermore, the morning meeting was beneficial because it facilitated communication within the ICU as *"it is easy – effective transmission of knowledge*" (doctor 1). The meetings facilitated work by bringing attention to what was going to happen during the day. For example, *"… the pharmacist can start ordering specialist drugs if you need them …"* (manager 1).

One benefit of the escalation plan was that it created a plan for how to operate when work was getting hectic since "*… it's made us more aware of forward planning and being proactive"* (manager 1). One example was when patients were transferred to a neighbouring hospital in accordance with the plan, another is that it increased the ICU consultants' authority to say no to elective surgeries when the ICU was under pressure.

Enabling factors

That the morning meetings are the first activity of the day and are held with all staff together, enabled its continuation. It was "*… the only point in time you can have everybody to sit down"* (manager 1). The morning meetings were beneficial from a human resources perspective because they ensured that *"people come to work on time"* (doctor 3). The visually displayed feedback of the colour coded bed status was thought to be beneficial. One

participant reported that *"it's easier to do it [record bed status] because we are doing it in a handover meeting in the morning"* (doctor 2). The implementation worked well for a while because *"everybody was on the right page"* (nurse 1), and *"… it worked because it was something everyone was willing to try" (*doctor 4).

Factors adversely affecting sustainability

Management of operations

Most participants reported that the pressure for elective surgeries has increased over the last five years and that there is a need for more resources to be allocated for the ICU. For example, *"there's a massive push to meet our NEST [National Elective Surgery] targets and we are behind …"* (doctor 1). And *"If there's no more money for ICU then we just have to accept that there's going to be more cancellations and longer delays, that's reality"* (manager 3).

Some participants expressed that there were hierarchical barriers that created a negative workplace culture, *"…there are good leaders and there are bad leaders and there are good leaders that help people progress... Then there are some people that just put barriers in front of people …"* (nurse 2). All but two participants believed that the leadership do not understand the work done by frontline staff. One nurse expressed this as *"I think people are actually considering themselves a number"* (nurse 2).

Lack of communication and understanding

A lack of transparency about how nurses were rostered onto shifts was expressed, "*there has been loss of transparency about the staffing that we do have in the unit… and that actual discussion about where we are, has been lost"* (doctor 1). Furthermore, there was a discrepancy between how nurses and doctors view the number of available beds. The nurses thought they had the capacity to fill up the ICU with elective cases, by increasing the nurse staff with flex time, whereas the doctors wanted to keep beds free in anticipation of emergency cases. The communication problems also extended outside the ICU resulting in communication problems with other wards and up to management level. For example, *"They have different booking staff down there that don't really know what goes on up here "*(nurse 1*)*, and *"…ICU need to speak the same language with the executive and surgical service group managers. We are not speaking the same language"* (doctor 2). It was also reported

For peer review only

that some staff are unfamiliar with the escalation plan. Bed status was only visual at morning meetings and therefore *"there is no eyeballing of that"* (doctor 1) which makes it easier to forget. On occasion, the staff didn't understand the point of the escalation plan, "*So the numbers are actually useless. The colour code is useless. It's only for internal use."* (doctor 2).

Plans not working as intended

Over the five years since it was introduced, the use of the escalation plan has changed. There are several examples of when the staff member needs to go the extra length to perform their duties *"I can't count the number of times I have brought my food to the bedside to eat"* (nurse 4). Some participants expressed that the escalation plan wasn't comprehensive enough and that it should include a plan for how to respond to emergency admissions.

Bed status was not filled out in the afternoon as regularly as was intended *"because busy-ness, and the setting makes it easier to forget"* (doctor 2). Several examples were given when the colour coding system did not work as intended, "*it's called green when we should be amber, it's called amber when we should be red*" (doctor 3), and *"there was no major result from"* escalating it up to management level (manager 2).

High workload predisposes for a workplace with a negative culture where *"Everyone's glum. There's no smiley. No happiness. No nothing and everything goes wrong."* (nurse 2), and *"… we couldn't bring the patient to ICU because there was no nurse to look after the patient. … I actually took time off sick, stress leave…"* (doctor 4).

Reality of clinical work

Lastly, some factors emerged over the years after the intervention that the escalation plan either didn't foresee or was unable to accommodate. For example, the introduction of electronic medical records, and delayed ward discharges. A common problem at ICUs is large variations in workload, and they are *"… either flooded or dry as we never get that rhythm happening for any period of time"* (manager 2).

A common view was that the reason for the escalation plan not working was that there were not enough nurses and that "*they're not always available when you need them."* (doctor 4). A

For peer review only

high staff turn-over rate with many newly recruited nurses was mentioned as detrimental for the work in the ICU "*we struggle, because we started to take graduates as well. They are only doubled up for a number of weeks and then we've got critically ill patients. That takes a lot more of the float nurse's time…"* (nurse 4).

Discussion

This study is a five-year follow-up study which gives insight into the long-term effect after implementation of an ICU patient flow intervention. To investigate if changes have been sustained, the evaluation should be done at least one year after the intervention.[1, 10] The intervention in 2014 in itself has continued to exist (the morning meeting, the bed status registration and the escalation plan), but the combined positive effects of the implementation have not been sustained over time. More elective surgeries are cancelled due to the unavailability of ICU beds from the second post-intervention year. The increase of cancelled elective surgeries is not explained by a higher bed occupancy or increase in the average LoS at the ICU. However, planned elective surgeries have increased and HPPD has significantly increased. This indicates that both demand for ICU post-surgical beds and the workload for the nurses has increased and that the increased pressure for elective surgeries that the participants expressed in the interviews was indeed true. The high variability of surgery cancellation rates a few years after the implementation are indicating a non-functioning process.

A major finding in the study is the lack of communication within ICU and between ICU and other departments, within the microsystem and between the micro- and mesosystems. Healthcare can be described as a complex adaptive system (CAS) with micro-, meso- and macrosystems. [11, 12] Microsystems are the smaller units that are formed around the patient at the point of care. [13, 14] The different microsystems are connected to and affect each other, through the mesosystem. In CAS the communication between components affects system performance; for risk management the micro- and mesosystems have to continually coordinate their responses to adapt to changing conditions.[15] The reported deficiencies in communication within ICU and between ICU and other departments may hence be contributing to that the positive effects of the implementation were not sustained over time.

For peer review only

Workplace culture issues adversely affected sustainability in several ways. For example, discrepancies were found between how nurses and doctors saw the availability of ICU beds and in that there were hierarchical barriers, this combined can create a negative workplace environment. A three-year follow-up study by Greenhalgh et al. found that good interrelations in the work environment are important for sustaining implementation.[16] They stated that when there is a lack of transparency with decision-making and workways, the communication is lost, and the microsystems cannot co-operate in harmony, which accords with our findings.

The multidisciplinary meetings have more positive effects associated with them than the escalation plan. That the morning meetings are always at the same time and in the same room is habit-forming, which can be an important element in sustaining change.[17] Several aspects of the meeting, such as the opportunity to review patients and communicate plans, support daily work in the ICU. As was also noted in the original study, the morning meeting facilitates coordination of the workday for several different professionals at the same time and reinforces teamwork.[6] These positive effects of the meetings improve the conditions within the ICU and could explain why they have become an integrated part of daily work.[18] Even though negative for the cancellation rate, one of the positive effects of the escalation plan, is that it increased the staffs' authority to cancel planned surgery, thus empowering the microsystem, which was also expressed in the original study.[6]

Even though most of the participants worked at the ICU during the implementation in 2014, there seems to have been a lack of understanding of the necessity with the escalation plan. The original study found that there was no consistent agreement on the purpose of the plan,[6] and this could explain why the commitment and dedication to the escalation plan has been lost over the years. If the benefits of the intervention are not readily perceived by the staff the chance of sustainability is lower.[7] For improvement leaders, management or engaged clinicians, it is important to be able to explain the purpose of the intervention, and to support knowledge and understanding among staff for long-term good quality outcomes.[7, 19] Furthermore, Staines et al. noted in a study of the long-term success of quality work in a healthcare organisation the importance of leadership commitment to improvement work and support to front-line staff work with improvements.[20] Thus, continuous follow-up by management to reinforce commitment is needed for a change to be fully accepted and firmly established at the workplace.

For peer review only

Several participants thought that the escalation plan was not sufficiently comprehensive to cover all situations in the real clinical work, and that this were an obstacle to the implementation of the plan. However, when new processes are introduced in CAS, it can't be expected that they will stay the same since the conditions are always changing. That an intervention can be modified over time is influencing the extent of sustainability.[7] It is therefore important to acknowledge the difference between Work-as-Imagined (WAI) and Work-as-Done (WAD).[21] It might be that the escalation plan, being WAI, is not flexible enough for the dynamics in the actual work going on in the ICU (i.e. WAD). One example is situations reported when the escalation plan was not used to protocol since the bed status classification did not fit reality. The original study, however, highlighted how the plan was co-opted by staff members in different ways, according to their own needs, suggesting that the plan was sufficiently flexible when it was first implemented.[6] Some of that flexibility may have been lost when the understanding and knowledge about the rationale for the escalation plan was lost. Interventions must be able to adapt to the changing context of healthcare to continuously refine and improve interventions to ensure they can be sustained.[22]

Study limitations

Besides bed status registration, and the report that daily morning meetings are ongoing, there are no hospital data for an audit of compliance to all parts of the intervention in retrospect. Furthermore, in the regression analysis, the R^2 is small for most analyses which suggests that the independent variable is only explaining a part of the variance in the outcome variable. Thus, there may be other factors that also explain the variance in the number of cancelled surgeries, for example staff and policies changes not reported, or baseline increase in demand due to the aging population. These factors could not be evaluated within the scope of this study.

Conclusion

In this study, even though practices of the interventions have been sustained over time, the positive effects have not. Thus, there is no such thing as a 'fix and forget' solution for the implementation of interventions in complex adaptive systems as healthcare. This study highlights important implications for interventions to improve healthcare. Management commitment to support communication within and between microsystems, and to support

For peer review only

healthcare staff understanding of the underlying reasons for intervention, are necessary for success over time after the initial enthusiasm with a new intervention. Further studies on how this is achieved are warranted.

Author contribution

All authors contributed to the planning and design of the study; ER collected the study data, and made the first analysis and interpretation, which were then finalised together with AR, RC-W and EA. ER and AR drafted the manuscript; EA, RC-W, LDG, PL and AJ revised it critically for intellectual content and approved the final version of the paper.

Funding

This research received no specific grant from any funding agency in the public, commercial or not-for profit sectors.

Competing interest

PL and AJ were involved in development of the intervention. They were however not interviewed as part of the study and were not involved in data collection or analysis. The authors have no other competing interests to declare.

Patient consent form

Not required.

Ethics approval

The Human Research Ethics Committee at the studied hospital (HREC/2019/QTHS/59796).

Data sharing statement

No data are available except those uploaded as supplementary information. Study data cannot be obtained by a third party as a requirement of ethics approval.

References

1. Scheirer MA, Dearing JW. An agenda for research on the sustainability of public health programs. *Am J Public Health.* 2011;101(11):2059-2067. doi: 10.2105/AJPH.2011.300193

For peer review only

2. Mesner SA, Foster NE, French SD. Implementation interventions to improve the management of non-specific low back pain: a systematic review. *BMC Musculoskelet Disord*. 2016;17:258. doi:10.1186/s12891-016-1110-z [published 10 Jun 2016]
3. Buchanan D, Fitzgerald L, Ketley D, et al. No Going Back: A Review of the Literature on Sustaining Organizational Change. *International Journal of Management Reviews* 2005;7(3):189 - 205. doi: 10.1111/j.1468-2370.2005.00111.x.
4. Fixsen DL, Naoom SF, Blase KA, et al. (2005). Implementation research: A synthesis of the literature. Tampa, FL: University of South Florida, Louis de la Parte Florida Mental Health Institute, The National Implementation Research Network (FMHI Publication #231).
5. Martin GP, Weaver S, Currie G, et al. Innovation sustainability in challenging health-care contexts: embedding clinically led change in routine practice. *Health Serv Manage Res.* 2012;25(4):190–199. doi:10.1177/0951484812474246
6. Clay-Williams R, Blakely B, Lane P, et al. Improving decision making in acute healthcare through implantation of an intensive care unit (ICU) intervention in Australia: a multimethod study. *BMJ Open* 2019;9:e025041. doi:10.1136/bmjopen-2018-025041.
7. Scheirer MA. Is sustainability possible? A review and commentary on empirical studies of program sustainability. *Am J Eval.* 2005;26(3):320–347. doi:10.1177/1098214005278752
8. Kirby KK. Hours per Patient Day: Not the Problem, Nor the Solution. *Nurse Econ.* 2015;33(1):64-6.
9. Denzin NK, Lincoln YS. Strategies of qualitative inquiry. 4th edn. US. Sage, 2013.
10. Wiltsey Stirman S, Kimberly J, Cook N, et al. The sustainability of new programs and innovations: a review of the empirical literature and recommendations for future research. *Implement Sci.* 2012;7:17. doi:10.1186/1748-5908-7-17
11. Braithwaite J, Clay-Williams R, Nugus P, et al. Health care as a complex adaptive system. In Hollnagel E, Braithwaite J, Wears RL, eds. Resilient Health Care. Farnham, Surrey, England: Ashgate Publishing. 2013: 57-73.
12. Nelson EC, Godfrey MM, Batalden PB, et al. Clinical Microsystems, Part 1. The Building Blocks of Health Systems. *Jt Comm J Qual Patient Saf.* 2008;34(7):367-78. doi: 10.1016/S1553-7250(08)34047-1

For peer review only

13. Mohr JJ, Batalden P, Barach P. Integrating patient safety into the clinical microsystem. *Qual Saf Health Care.* 2004;13:ii34–ii38. doi: 10.1136/qshc.2003.009571
14. Johnson JK, Batalden PB. Educating health professionals to improve care within the clinical microsystem. McLaughlin and Kaluzny's Continuous Quality Improvement In Health Care. Burlington: Jones & Bartlett Learning; 2013.
15. Rasmussen J. Risk management in a dynamic society: a modelling problem. *Saf Sci.*1997;27(2-3):183-213. doi:10.1016/S0925-7535(97)00052-0
16. Greenhalgh T, Macfarlane F, Barton-Sweeney C, et al. "If we build it, will it stay?" A case study of the sustainability of whole-system change in London. *Milbank Q.* 2012;90(3):516–547. doi:10.1111/j.1468-0009.2012.00673.x
17. Fleiszer AR, Semenic SE, Ritchie JA, et al. An organizational perspective on the long-term sustainability of a nursing best practice guidelines program: a case study. *BMC Health Serv Res.* 2015;15:535. doi:10.1186/s12913-015-1192-6
18. Aston J, Shi E, Bullôt H, Galway R, Crisp J. Qualitative evaluation of regular morning meetings aimed at improving interdisciplinary communication and patient outcomes. *Int J Nurs Pract.* 2005;11(5):206-13. doi:10.1111/j.1440-172X.2005.00524.x
19. Sandberg J, Targama A. Managing understanding in organizations. London: SAGE; 2007.
20. Staines A, Thor J, Robert G. Sustaining improvement? The 20-year Jönköping quality improvement program revisited. *Qual Manag Health Care.* 2015;24(1):21–37. doi:10.1097/QMH.0000000000000048
21. Clay-Williams R, Hounsgaard J, Hollnagel E. Where the rubber meets the road: using FRAM to align work-as-imagined with work-as-done when implementing clinical guidelines. *Implement Sci*. 2015;10:125. doi:10.1186/s13012-015-0317-y
22. Chambers DA, Glasgow RE, Stange KC. The dynamic sustainability framework: addressing the paradox of sustainment amid ongoing change. *Implementation Sci.* 2013;8:117. doi:10.1186/1748-5908-8-117

Table 1: Surgeries with post-operative ICU recovery, planned and cancelled

	Planned elective	Cancelled surgeries

	surgeries requiring an ICU bed, n	due unavailability of an ICU bed, n (%)
First year after intervention (Dec 2014 – Nov 2015)	681	8 (1.17)
Year 2 (Dec 2015 – Nov 2016)	580	18 (3.10)
Year 3 (Dec 2016 – Nov 2017)	560	27 (4.82)
Year 4 (Dec 2017 – Nov 2018)	736	27 (3.67)
Year 5 (Dec 2018 – Nov 2019)	742	33 (4.45)

Table 2: Themes and subthemes found in the analysis in the interviews

Themes	Sub-themes
Sustained benefits of implementation	Positive experiences
	Enabling factors
Factors adversely affecting sustainability	Management of operations
	Lack of communication and understanding
	Plans not working as intended
	Reality of clinical work

Figure Legends:

Figure 1: Proportion of planned surgeries that were cancelled due to no available ICU beds per quarter, p-chart. Q1 is the first quarter in the study period, December 2014 – February 2015.

Figure 2: Changes in ICU demand or workload over the study period.
2a) Hours per patient day (HPPD) a measure of workload for nurses, per date.
2b) Occupancy at ICU, per date.
2c) Average Length of stay at ICU (LoS), per month.
2d) Monthly number of planned surgical admissions to ICU, per month.

For peer review only

Figure 1: p-chart bed cancellations per quarter

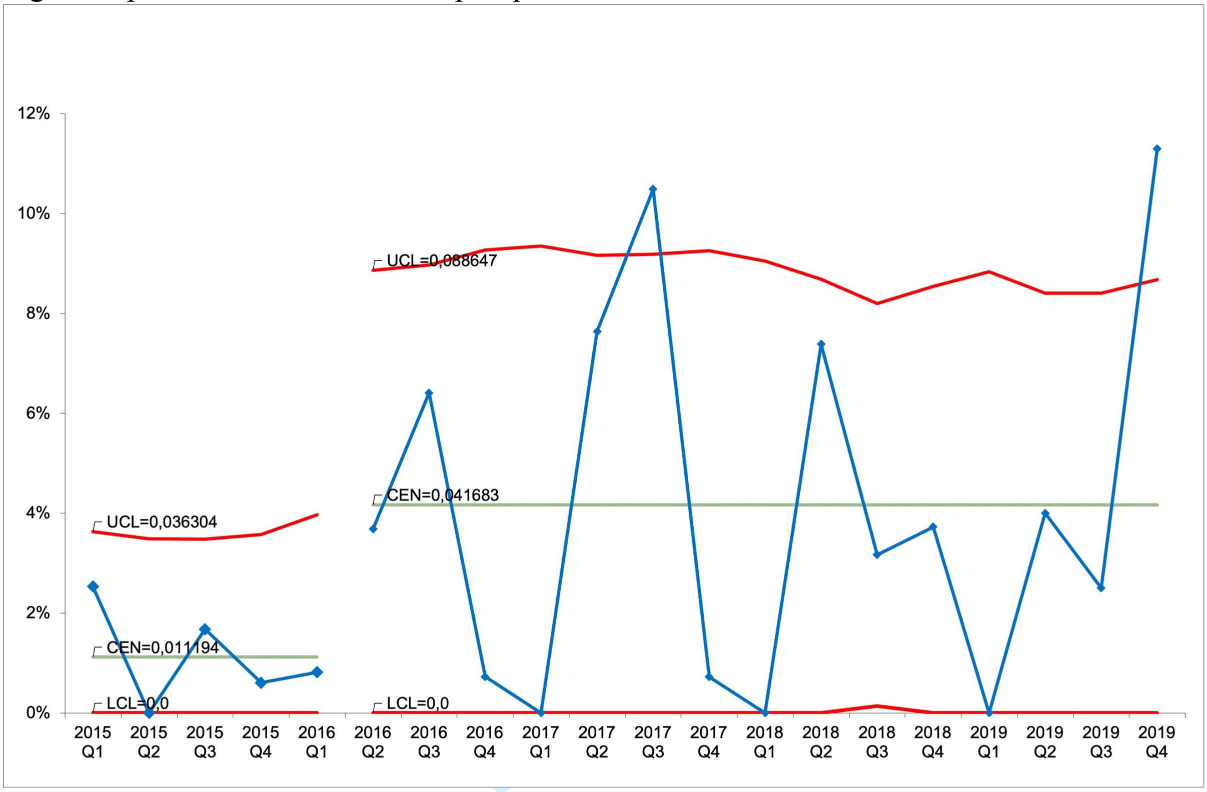

Legend: Proportion of planned surgeries that were cancelled due to no available ICU beds per quarter, p-chart. Q1 is the first quarter in the study period, December 2014 – February 2015.

Figure 2: Changes in ICU demand or workload over the study period.

2a) Hours per patient day (HPPD) a measure of workload for nurses, per date.

2b) Occupancy at ICU, per date.

2c) Average Length of stay at ICU (LoS), per month.

2d) Monthly number of planned surgical admissions to ICU, per month.

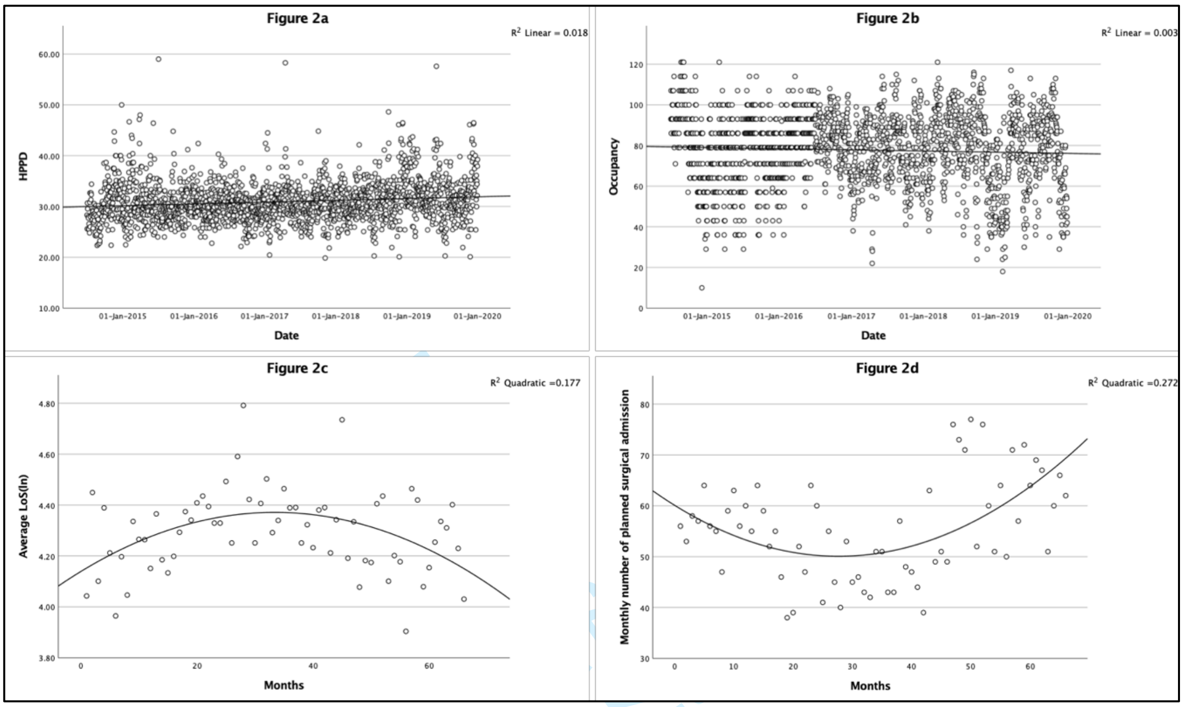

Appendix A: Interview Schedule

The information collected will be demographic information and responses to the following questions.

A: The ICU Escalation plan and morning handover meeting implementation

Do you know of the ICU Escalation plan and morning handover meetings that was implemented 2014?
Were you involved in the implementation process?
If so – what was your role?
What is your experience of the morning handover meetings (if relevant)?
What is your experience of the ICU Escalation plan (if relevant)?
Can you describe an episode when the ICU Escalation plan was activated (if relevant)?

B: ICU Resource allocation processes

Please describe how beds and resources are allocated in the ICU.
What positions have an input into these decisions?
What are the factors involved?
Does the process change as the ICU becomes busier?

C: Work practice changes

Questions to the hospital management group.
How does your work at the hospital involve the ICU?
Tell me how the ICU impacts on your daily work routine.
Are you aware of any change to your work practice due to the Escalation plan implementation?
Are you aware of any change to the work practices of other staff as a result of the initiatives?

Questions to ICU staff.
Are you aware of any change to your work practice due to the morning handover meetings?
Are you aware of any change to your work practice due to the Escalation plan implementation?
Are you aware of any change to the work practices of other staff as a result of the initiatives?

D: Job satisfaction

How do you feel about coming to work each day, particularly on days with heavy ICU involvement?
Was there any impact of the initiatives on your job satisfaction?
Did the initiatives make any difference to the days when ICU resources where stretched?
Do you feel patient care was improved?

For peer review only

E: Questions on the sustainability of the ICU Escalation Plan and daily morning meeting. Two different sets of questions to be used depending on the results of the analysis of the quantitative data.

I) *If the analysis of data shows that the improvement has sustained over time*
To your opinion – why has the benefits of the invention sustained over time?
What has been the key factors?
Who has been the key players? What did they do to help the success?
What suggestions do you have for the work with optimizing flow of patients through ICU?

II) *If the analysis of data shows that the improvement has not sustained over time*
To your opinion – why has the benefits of the invention not sustained over time?
What has been the obstacles?
What could have been done differently?
What suggestions do you have for the work with optimizing flow of patients through ICU?

F: Demographic Information

This information will not be linked to participants name. A number will be given to be used as a code reference for analysis purposes.

1.Gender
2.Profession
3.Role at TTHS
4.Time since qualification to practice his profession
5.Time in this organisation
6.Time in ICU

For peer review only

Appendix B: Code schedule

**Theme**	**Sub-theme**	**Code**	**Quotes**
Sustained benefits of implementation	Positive experiences	Facilitates communication within microsystem	Nurse 3: *"[…] as soon as you might say to one of the intensivists, where are we, and you can say, red, they know exactly the situation we're in. So, it's a good, quick identification."*
		Work-as-imagined reconciles work-as-done	Nurse 2: *"The doctors have been really good, so then they won't admit or […] they wouldn't admit high dependency patients. […] They've been very good supporting the staff and they've been - at one stage I remember them actually having to transfer to The Mater or something patients that had come into ED (emergency department), because we couldn't take them here, because he had no beds."*
		Improves teamwork	Manager 1: *"I think the morning meetings have made everybody more aware and tried to collaborate as a team approach, and I think that's really important."*
	Enabling factors	Morning meeting setting benefits	Manager 1: *"[…] it is continuous but that is the only point in time you can have everybody to sit down."*
		Everyone was invested	Doctor 4: *"Well, it worked because it was something everyone was willing to try."*
Factors adversely affecting sustainability	Management of operations	Leadership not understanding micro level work	Doctor 3: *"I'm going to be harsh here, a lot of the senior nursing staff who are going to be making those decisions are not there at ten o'clock at night or two in the morning, having to actually wear the brunt, the consequences of those decisions."*

For peer review only

		Hierarchical barriers to improvement	Nurse 2: *"[…] sometimes there are good leaders and there are bad leaders and there are good leaders that help people progress and things like that. Then there are some people that just put barriers in front of people. There are some people that just like arguing for the sake of arguing, which doesn't do the unit very good."*
		Pressure for elective surgeries	Doctor 1: *"There's a massive push to meet our NEST targets and we are behind and our service group is, I think, the only one who ended up in the red financially because we didn't meet our targets."*
		Lack of ICU resources	Manager 3*: "[…] if we have the same amount of ICU beds next year, the number of elective surgeries we cancel will just increase. If there's no more money for ICU then we just have to accept that there's going to be more cancellations and longer delays, that's reality."*
	Lack of communication and understanding	Lack of knowledge about implementation	Nurse 3: *"I don't know whether the new registrars and the new shift coordinators have a full grasp of the traffic light system."*
		Micro doesn't understand the escalation plan	Doctor 2: *"At the borderline state it doesn't do a lot. When it's really severe, when reach beyond red, which is very rare, it could be useful."*
		Lack of communication and discrepancy within microsystem	Doctor 1: *"[…] there has been loss of transparency about the staffing that we do have in the unit. We are often told oh, don't worry about that or we'll get staff, or you don't need to know that or yep, so I think that the lack of transparency and that actual*

For peer review only

			discussion about where we are, has been lost."
		Lack of communication and discrepancy between microsystems	Nurse 1: *"They have different booking staff down there that don't really know what goes on up here and so they'll just have a surgeon that wants to operate. He's got a theatre, so they'll say yes to anything not really realising that those patients do need to come to an intensive care unit […]."*
		Lack of communication between micro and meso	Doctor 2: *"[…] ICU need to speak the same language with the executive and surgical service group managers. We are not speaking the same language."*
	Plans not working as intended	Misuse of traffic light	Manager 2: *"There's a lot of disagreement, I think, around even though we've all agreed on the criteria for red, there's still, on the day of, flexibility in the thinking that yes you can get that heart done or yes you can get that elective surgery case in or no you can't, that sort of give and take."*
		Use of escalation plan has changed over time	Doctor 3: *"The process at the beginning was fine. It's been progressive reinterpretation that's given it grief."*
		Extra effort needed	Nurse 4: *"There are many days where we haven't get breaks. We just don't get breaks. We can't get staff to the bedside to help with turns […]. You should have two nurses to turn them. You don't do that. You can't get them. People get cranky with each other because they're not getting their breaks. We get around things by - often - I can't count the number of times I have brought my food to the bedside to eat[…]."*

For peer review only

		Consequences of over-loaded ICU	Doctor 4: *"[…] we couldn't bring the patient to ICU because there was no nurse to look after the patient. That actually made me go sick. […]I actually took time off sick, stress leave, and developed shingles, reactivation of chickenpox due to the stress."*
		Implementation not comprehensive enough	Manager 2: *"[…] it's very difficult to draft for everything that could happen and it's not black and white"*
	Reality of clinical work	Disturbance in patient flow	Manager 4: *"And then there's days where there's, potentially, patients who could go to the ward, and there's nowhere to send them to."*
		Increased workload for nurses	Nurse 2: *"I think the workload's increased for the nurses."*
		Staffing problem	Nurse 2: *"We have more new nurses. […] For transition. Like, they've just gone to uni. They've had no ward - they've had no experience whatsoever and they're coming up here, so that takes a lot of time away."*
		New clinical and administrative processes	Doctor 2: *"So it's actually worse in the last year with the EMR (electronic medical record) because it increased our workload."*
		Natural variation	Doctor 1: *"[…] January and February are classically - and I think it happens every year, really slow months. Then around August is always a peak. It's around flu season and more people have MIs and things like that but it seems to be a - not*

For peer review only

			necessarily predictable but a constant variation."

For peer review only

COREQ (COnsolidated criteria for REporting Qualitative research) Checklist

A checklist of items that should be included in reports of qualitative research. You must report the page number in your manuscript where you consider each of the items listed in this checklist. If you have not included this information, either revise your manuscript accordingly before submitting or note N/A.

Topic	Item No.	Guide Questions/Description	Reported on Page No.
**Domain 1: Research team and reflexivity**			
Personal characteristics			
Interviewer/facilitator	1	Which author/s conducted the interview or focus group?	5
Credentials	2	What were the researcher's credentials? E.g. PhD, MD	5
Occupation	3	What was their occupation at the time of the study?	5
Gender	4	Was the researcher male or female?	5
Experience and training	5	What experience or training did the researcher have?	5
Relationship with Participants:			
Relationship established	6	Was a relationship established prior to study commencement?	6
Participant knowledge of the interviewer	7	What did the participants know about the researcher? e.g. personal goals, reasons for doing the research	6
Interviewer characteristics	8	What characteristics were reported about the inter viewer/facilitator? e.g. Bias, assumptions, reasons and interests in the research topic	5
**Domain 2: Study design**			
Theoretical framework			
Methodological orientation and Theory	9	What methodological orientation was stated to underpin the study? e.g. grounded theory, discourse analysis, ethnography, phenomenology, content analysis	6
Participant selection			
Sampling	10	How were participants selected? e.g. purposive, convenience, consecutive, snowball	6
Method of approach	11	How were participants approached? e.g. face-to-face, telephone, mail, email	5-6
Sample size	12	How many participants were in the study?	6
Non-participation	13	How many people refused to participate or dropped out? Reasons?	N/A
Setting			
Setting of data collection	14	Where was the data collected? e.g. home, clinic, workplace	6
Presence of non-participants	15	Was anyone else present besides the participants and researchers?	6
Description of sample	16	What are the important characteristics of the sample? e.g. demographic data, date	6, 7-8
Data collection			
Interview guide	17	Were questions, prompts, guides provided by the authors? Was it pilot tested?	6
Repeat interviews	18	Were repeat inter views carried out? If yes, how many?	N/A
Audio/visual recording	19	Did the research use audio or visual recording to collect the data?	6
Field notes	20	Were field notes made during and/or after the inter view or focus group?	N/A
Duration	21	What was the duration of the inter views or focus group?	7
Data saturation	22	Was data saturation discussed?	8
Transcripts returned	23	Were transcripts returned to participants for comment and/or correction?	N/A

For peer review only

Topic	Item No.	Guide Questions/Description	Reported on Page No.
**Domain 3: analysis and findings**			
Data analysis			
Number of data coders	24	How many data coders coded the data?	6
Description of the coding tree	25	Did authors provide a description of the coding tree?	8
Derivation of themes	26	Were themes identified in advance or derived from the data?	6, 8
Software	27	What software, if applicable, was used to manage the data?	N/A
Participant checking	28	Did participants provide feedback on the findings?	6
Reporting			
Quotations presented	29	Were participant quotations presented to illustrate the themes/findings? Was each quotation identified? e.g. participant number	8-11
Data and findings consistent	30	Was there consistency between the data presented and the findings?	Yes
Clarity of major themes	31	Were major themes clearly presented in the findings?	8-11
Clarity of minor themes	32	Is there a description of diverse cases or discussion of minor themes?	8-11

Developed from: Tong A, Sainsbury P, Craig J. Consolidated criteria for reporting qualitative research (COREQ): a 32-item checklist for interviews and focus groups. *International Journal for Quality in Health Care*. 2007. Volume 19, Number 6: pp. 349 – 357

For peer review only
